# Aryl azopyrroles as visible light photoswitchable TRPA1 ligands

**DOI:** 10.1039/d5sc05070g

**Published:** 2025-09-16

**Authors:** Lisa C. Dollhopf, Jordan A. Munos, Kai Y. Zheng, Rui Xin Tao, Peter R. Haycock, Philip J. Parsons, Randall T. Peterson, Pui-Ying Lam, Matthew J. Fuchter

**Affiliations:** a Molecular Sciences Research Hub, Department of Chemistry, Imperial College London Wood Lane London W12 0BZ UK; b Department of Cell Biology, Neurobiology and Anatomy, Medical College of Wisconsin 53226 Milwaukee WI USA plam@mcw.edu; c Department of Chemistry, University of Oxford, Chemical Research Laboratory 12 Mansfield Road Oxford OX1 3TA UK matthew.fuchter@chem.ox.ac.uk; d Department of Pharmacology and Toxicology, University of Utah Salt Lake City UT USA

## Abstract

Access to high quality optochemogenetic tools will enable precise interrogation of the complex biology of diverse cell types, allowing temporally and spatially controlled manipulation of cell autonomous functions. This level of control is expected to yield deeper mechanistic insights and foster the development of targeted strategies and molecular interventions for translational photopharmacology. Recent years have demonstrated the exciting potential of heteroaromatic azoarene photoswitches, particularly to tune azo photoswitch performance. However, strategies to integrate such motifs into photopharmacological ligands remain underdeveloped. Leveraging a previously developed screening assay, we report the discovery of a robust aryl azopyrrole ligand for the TRPA1 channel, TRPswitch-C. TRPswitch-C provides a red-shifted chemotype to address this channel, allowing optical control in live zebrafish larvae in a controllable fashion. Not only does this study expand the tool kit for optochemogenetic experiments using TRPA1, but also advocates the further exploration of heteroaromatic azoarenes in photopharmacology.

## Abstract 2

We report the discovery of a robust aryl azopyrrole ligand for the TRPA1 channel, TRPswitch-C. TRPswitch-C provides a red-shifted chemotype to address this channel, allowing optical control in live zebrafish larvae in a controllable fashion.

## Graphical abstract

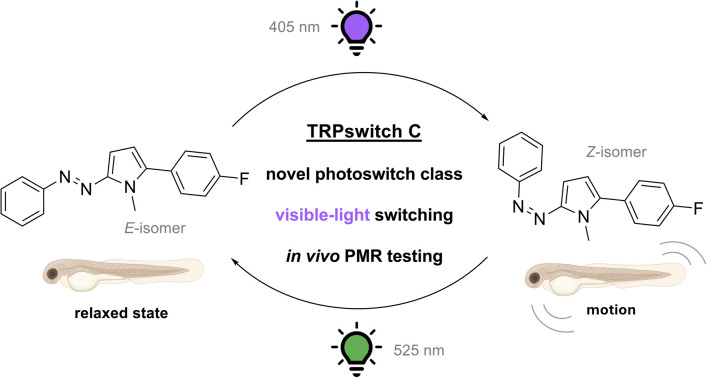

## Introduction

Photopharmacology using molecular photoswitches represents a powerful way to endow bioactive ligands with reversible light addressability. By using light to interconvert the structural states of the photoswitch, the bioactivity of the corresponding photopharmacological ligand can be modulated as a function of the wavelength.^1–5^ The most common photoswitch for photopharmacology, by far, is azobenzene. However, recent studies have shown that superior photoswitch performance can be achieved using heteroaromatic azo switches.^6,7^ Despite this advance, heteroaromatic azo photoswitches remain relatively underexplored in photopharmacology.^8^

The discovery of photopharmacological ligands mostly relies on modification of a known non-photoswitchable ligand, using one of two complementary approaches called “azologization” and “azo extension”.^1,9^ The former seeks to replace elements of the ligand core structure with an isosteric azo photoswitch,^9^ while the latter adds an azo photoswitch to the periphery of the ligand. While these are powerful and effective ways to develop photopharmaceuticals, they are reliant on preexisting non-switchable ligands. *De novo* discovery of photopharmacological ligands remains poorly developed. In a rare example, we previously screened a curated library of photoswitches in a phenotypic zebrafish assay and discovered the first photoswitchable ligands of the TRPA1 channel, TRPswitch-A and B.^10^ A more recent example of *de novo* discovery of a different photoswitch scaffold, Plinabulin and analogues as tubulin-binding hemipiperazine switches, has been reported by Kirchner *et. al*.^11^ Beyond validation of screening as an effective means for photopharmacological ligand discovery, this work resulted in the identification of light-addressable tool molecules that have further enabled interrogation of TRPA1 biology^12^ and hold potential in other chemo-optogenetic experiments in large neurons, such as those in the nucleus gigantocellularis,^13^ where a large depolarization current is needed.

A notable omission from our initial screening library was heteroaromatic azo photoswitches; our reported azylazopyrazole ligand TRPswitch-B was a subsequently optimized version of our initial azobenzene screening hit, TRPswitch-A.^10^ Here we use our assay to identify an aryl azopyrrole ligand of TRPA1, which represents a distinct chemotype over TRPswitch-A/B. This ligand can be robustly addressed in live zebrafish larvae using visible light and represents the first azopyrrole used in photopharmacology. We believe this work further advocates new discovery methodologies for photopharmacology and the strengthens the case for heteroaromatic azo photoswitches in this area.

## Results and discussion

By screening small libraries of heteroaromatic switches, accessed through our previous structure–property studies,^14–16^ and using our previously reported light induced motion response assay,^10^ we identified aryl azopyrroles as a promising chemotype to control movement in zebrafish larvae. The electron-rich pyrrole ring results in red-shifting of the absorption bands for such switches, which in principle allows for visible light addressability.^14,15^ However, aryl azopyrroles can be prone to rapid thermal *Z*–*E* isomerization, especially in the presence of water,^15^ which has limited their wider use in photoswitch applications.^17^ Nonetheless, TRPswitch-C, which is an effective modulator of TRPA1 (see below), was also found to be a robust aryl azopyrrole photoswitch, with an extended thermal half-life for the *Z* isomer.

**Scheme 1:**
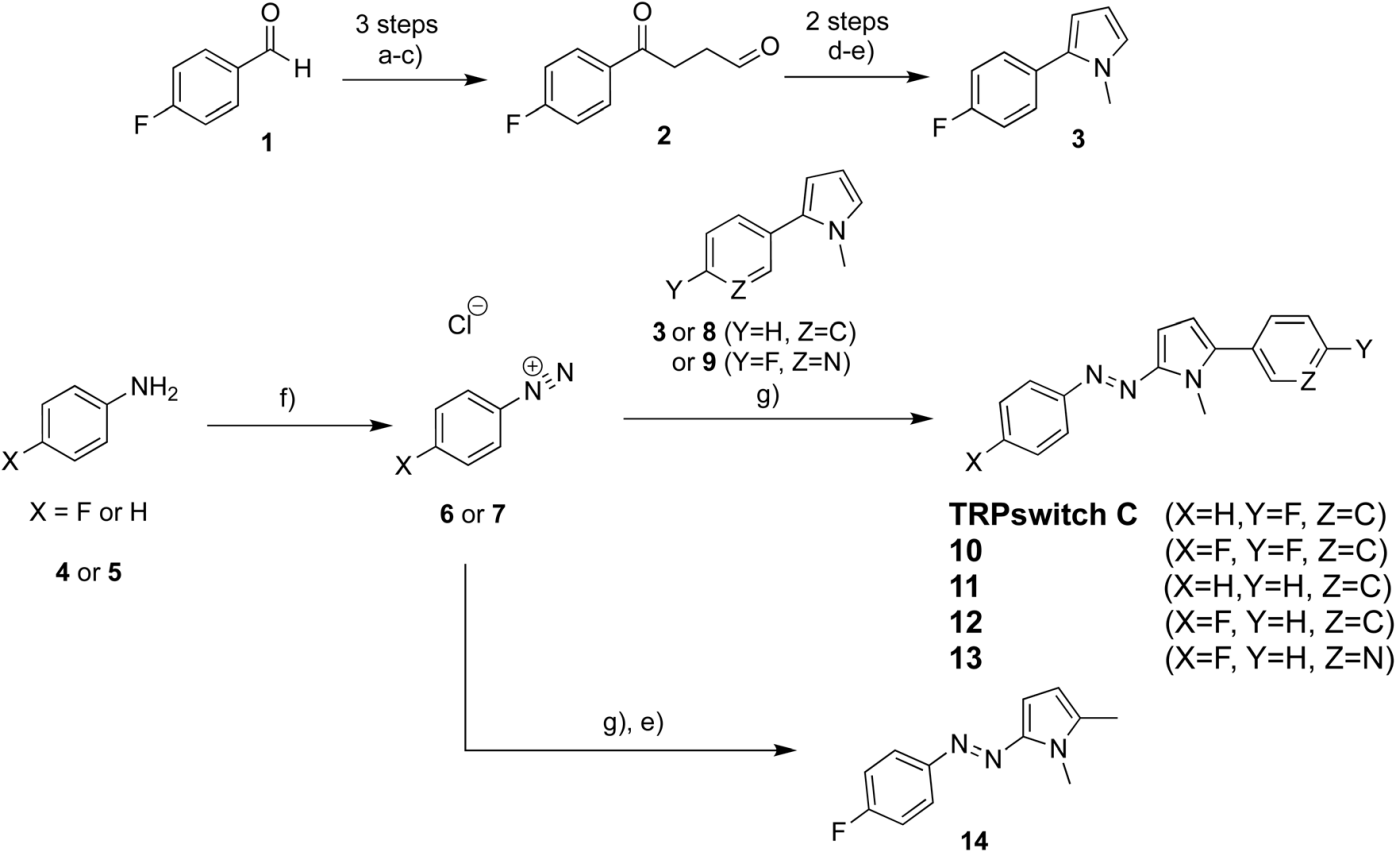
Reagents and conditions. (a) Allyl magnesium chloride, THF, 0 °C to room temperature, 3 h. (b) 2 M BH_3_·DMS in THF, then aq. NaOH and H_2_O_2_, 0 °C to room temperature, 5 h. (c) (COCl)_2_, DMSO, NEt_3_, DCM, −78 °C to room temperature, 1.5 h, 33% over 3 steps. (d) NH_4_OAc, EtOH, reflux, 1.5 h, 85%. (e) Iodomethane, NaH, THF, 0 °C to 60 °C, 1.5 h, 40–61%. (f) NaNO_2_, 1 M HCl, MeOH, 0 °C, 30 min; then (g) pyrroles, NaOAc, EtOH, water, 0 °C, 1 h, 8–28%.

All azopyrrole photoswitches were synthesized *via* diazonium coupling between an aryl diazonium salt and a corresponding pyrrole (Scheme 1). Intermediate 2 was synthesized according to a known literature procedure.^18^ Briefly, 4-fluorobenzaldehyde 1 was reacted with allyl magnesium chloride, followed by a subsequent hydroboration-oxidation and Swern oxidation. This resulted in the desired aldehyde 2 in moderate yield (33% over 3 steps). Key pyrrole intermediate 3 was obtained through condensation of 2 with ammonium acetate, followed by N-alkylation of the generated pyrrole with NaH and iodomethane in THF (61% yield). Anilines 4 and 5 were first diazotized, followed by coupling with the respective pyrroles under basic conditions in an EtOH/water mixture. The final products TRPswitch-C and 10–13 were obtained in moderate yields (8–28% over 2 steps). Final compound 14 was obtained after subsequent N-methylation with NaH and iodomethane in THF.

**Fig. 1:**
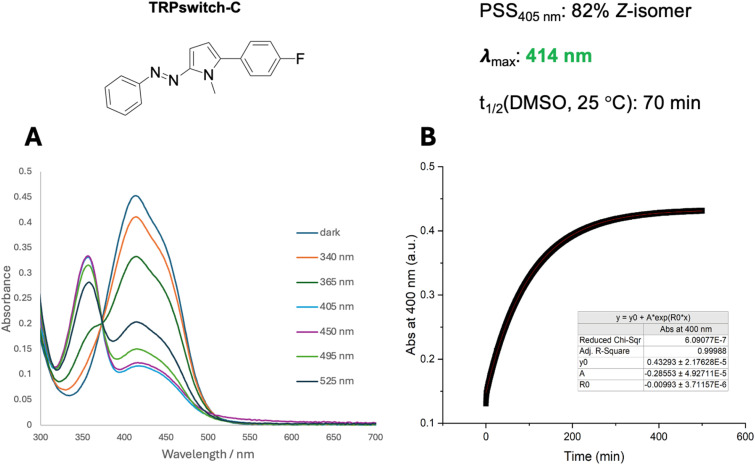
Photochemical characterization of TRPswitch-C. (A) UV-vis spectra of TRPswitch-C in DMSO (50 μM) at 25 °C were recorded before and after irradiation with LEDs of different wavelengths for 3 minutes. PSS ratios were determined according to the method described by Fischer.^19^ (B) Thermal isomerization kinetics of TRPswitch-C in DMSO (50 μM) at 25 °C. The absorbance at 400 nm was fitted to an exponential fit to determine the rate constant and thermal half-life of *Z*–*E* isomerization.

The UV/vis spectrum of TRPswitch-C is shown in Fig. 1A. TRPswitch-C showed a strong π–π* absorption band with a characteristic shoulder that was observed for all studied azopyrrole photoswitches. Irradiation with UV (340 and 365 nm) and visible light (405 and 450 nm) resulted in good *E* to *Z* isomerization of up to 82% in DMSO at room temperature. The absorption of these aryl azopyrrole switches are significantly red-shifted (*Z* isomer *λ*_max_ = 414 nm) in comparison to previously reported arylazopyrazole based TRPswitches.^10^ Due to the broad absorption bands, relatively poor *Z* to *E* photoswitching was observed (39% *E* isomer after irradiation with 525 nm light). A half-life of 70 minutes in DMSO was obtained from thermal isomerization kinetics experiments that measured the absorption at 400 nm of a previously irradiated *Z*-isomer rich sample (Fig. 1B).

**Fig. 2:**
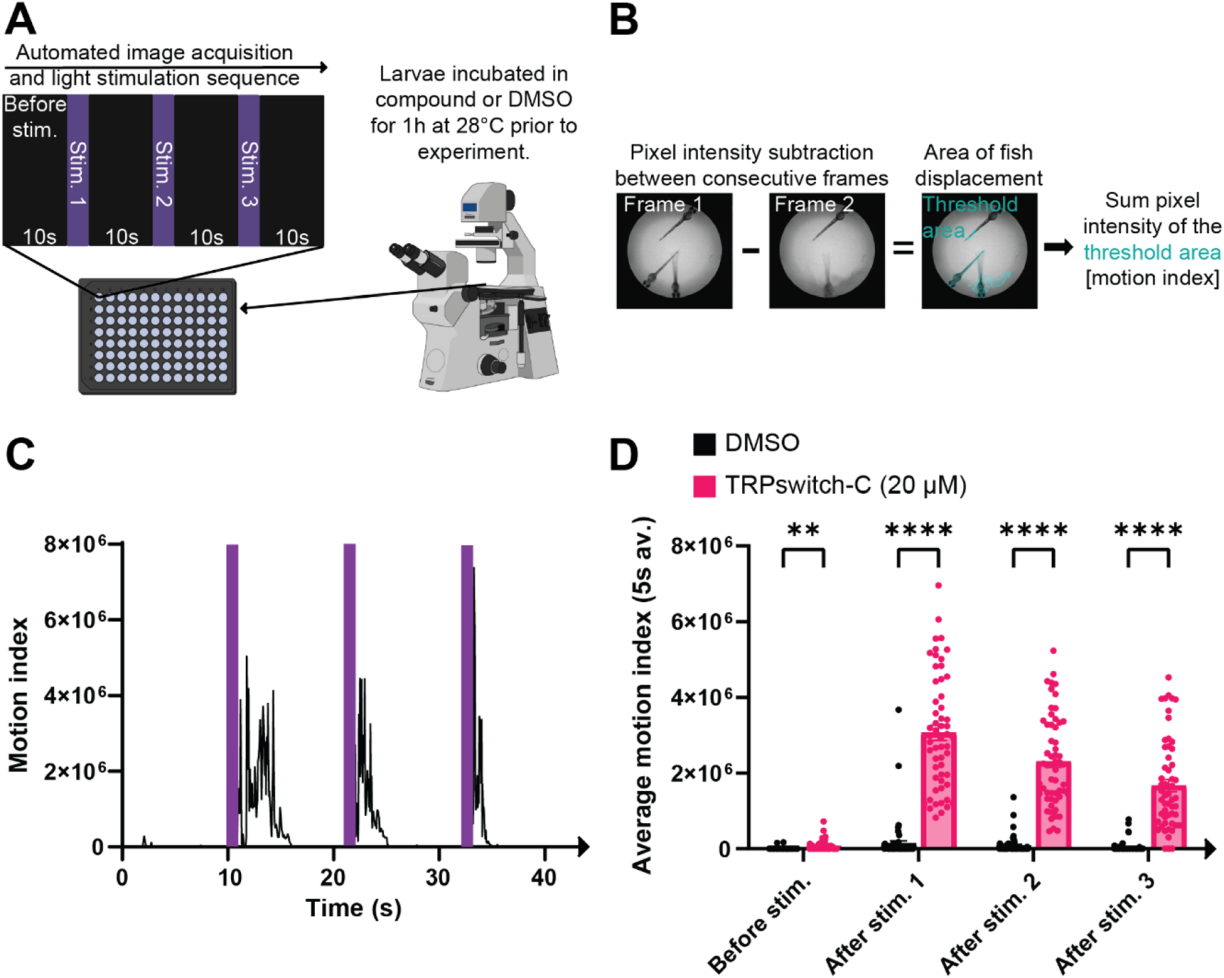
Properties of TRPswitch-C. (A) Schematic of the experimental setup for the light induced motion response assay. Compounds were screened in a 96-well plate using 3 day post fertilization (dpf) wild-type zebrafish (zf) larvae. Three 3dpf zf were placed into 1 well and compounds were added. A motorized inverted compound microscope and an automated light sequence were used. Hit compounds have a motion response after light stimulation when compared to DMSO controls. (B) Quantification of the motion response (see methods section for details). (C) Representative line graph of the zf behavioral response when exposed to 20 μM TRPswitch-C. Violet bars indicate when stimulation light is on (duration 1 s). (D) Quantification of the behavioral response of fish exposed the TRPswitch-C or DMSO before and after light stimulation. Each data point is a single well. Values are mean ± SEM. (*p*-value ** < 0.0021; **** < 0.0001) see SI Movie 1.

We assessed our aryl azopyrrole compounds in our previously reported motion response assay. In short, three 3 day post fertilization (dpf) wildtype (WT) zebrafish (zf) larvae were placed in a well of a 96 well plate and exposed to test compounds (20 μM) at 28 °C for 1 h in the dark prior to the experiment. At 3 dpf, zf larvae do not respond to a light stimulus, thus any light-induced motion response observed would be due to compound treatment. DMSO (1%) was used as a negative control for all experiments. Each well was individually stimulated with three 1 s pulses of stimulation light (Fig. 2A). Throughout the light stimulation sequence, images were acquired without delay. The readout of the assay is a light induced motion response. We quantified the motion before and after light illumination with a custom script developed using NIS Elements GA3 software, thus allowing us to compare DMSO controls to compound treated wells (Fig. 2B). TRPswitch-C caused a robust motion response produced upon each light illumination when stimulated with violet light (Fig. 2C and D and SI Movie 1). The light induced motion response was not observed in larvae incubated in DMSO.

**Fig. 3:**
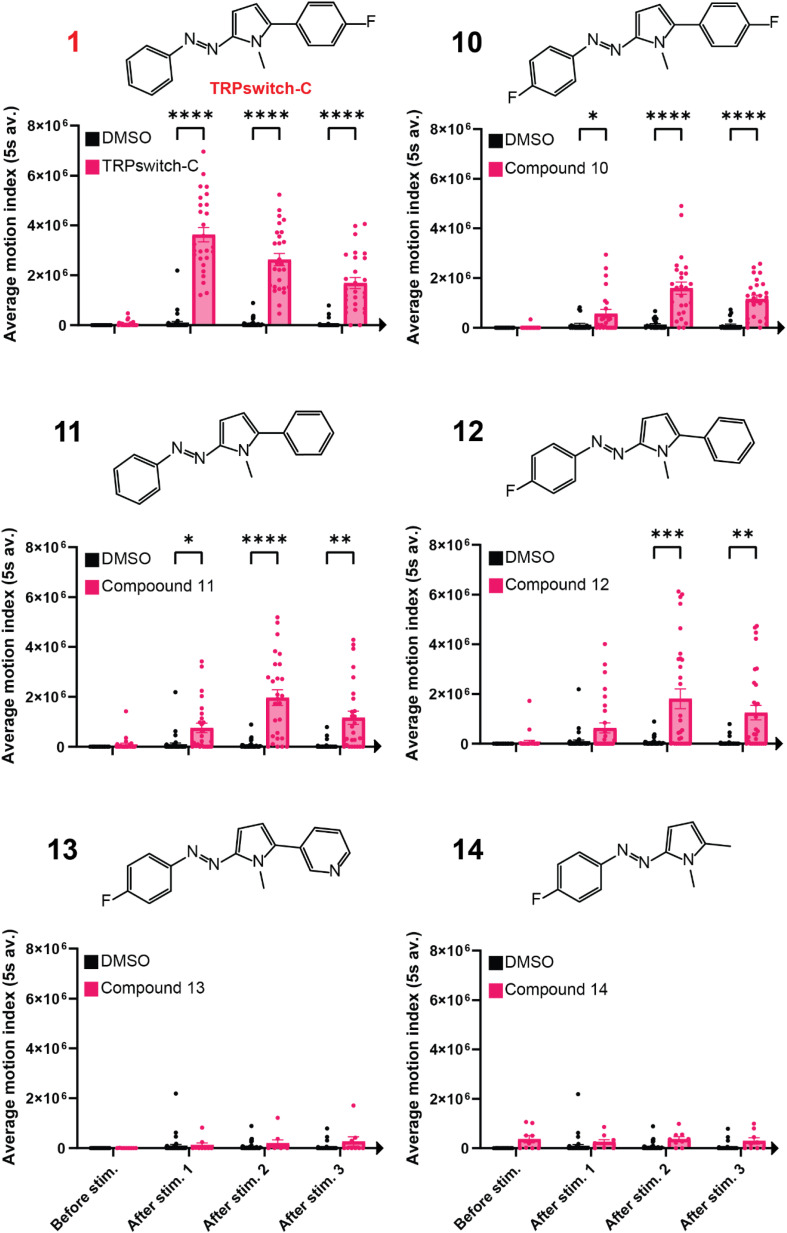
TRPswitch-C structure–activity relationship analysis. Structures for each compound are on top of the corresponding graph of the light induced motion assay result. 390 nm light was used for stimulation for all compounds except compound 10, which used 440 nm for stimulation. Data points are individual wells. Values are mean ± SEM. (*p*-value * < 0.0332; ** < 0.0021; *** < 0.0002; **** < 0.0001).

We sought to briefly survey the structure property relationships that were important for the activity of TRPswitch-C, particularly the importance of fluorine substitution and the need for an aromatic ring attach to the pyrrole unit. As such, we generated a series of derivatives (compounds 10–14) and characterized them using our motion response assay. Compound 10 contains a fluorine group in the para position on either benzene ring (Fig. 3) and undergoes *E*/*Z* isomerization most efficiently with 440 nm light. We observed reduced photoswitching and biological activity when 390 nm light was used for stimulation with compound 10. When 3 dpf WT zf were exposed to compound 10 there was an increase in motion after 440 nm light stimulation (Fig. 3) compared to DMSO controls, but less than that observed for TRPswitch-C. When both fluorines were removed from the compound (compound 11) the optimal wavelength of photoactivation becomes 390 nm. The motion response of 11 is similar to 10. When the F is removed from the para position on the right side of the compound (compound 12), the motion response is, again, similar to that of compounds 10 and 11. As such, it seems this scaffold is modest in its sensitivity to fluorine substitution, with TRPswitch-C having the optimum combination of those analogues we tested. To examine if different substitutions of the ring were tolerated, compounds 13 and 14 were synthesized. Compound 13 replaced the benzene ring with a pyridine ring while compound 14 removed the ring altogether. A lack of motion was observed with both 13 and 14 (Fig. 3) suggesting the benzene ring attached to the pyrrole to be critical for motion response in our assay.

**Fig. 4:**
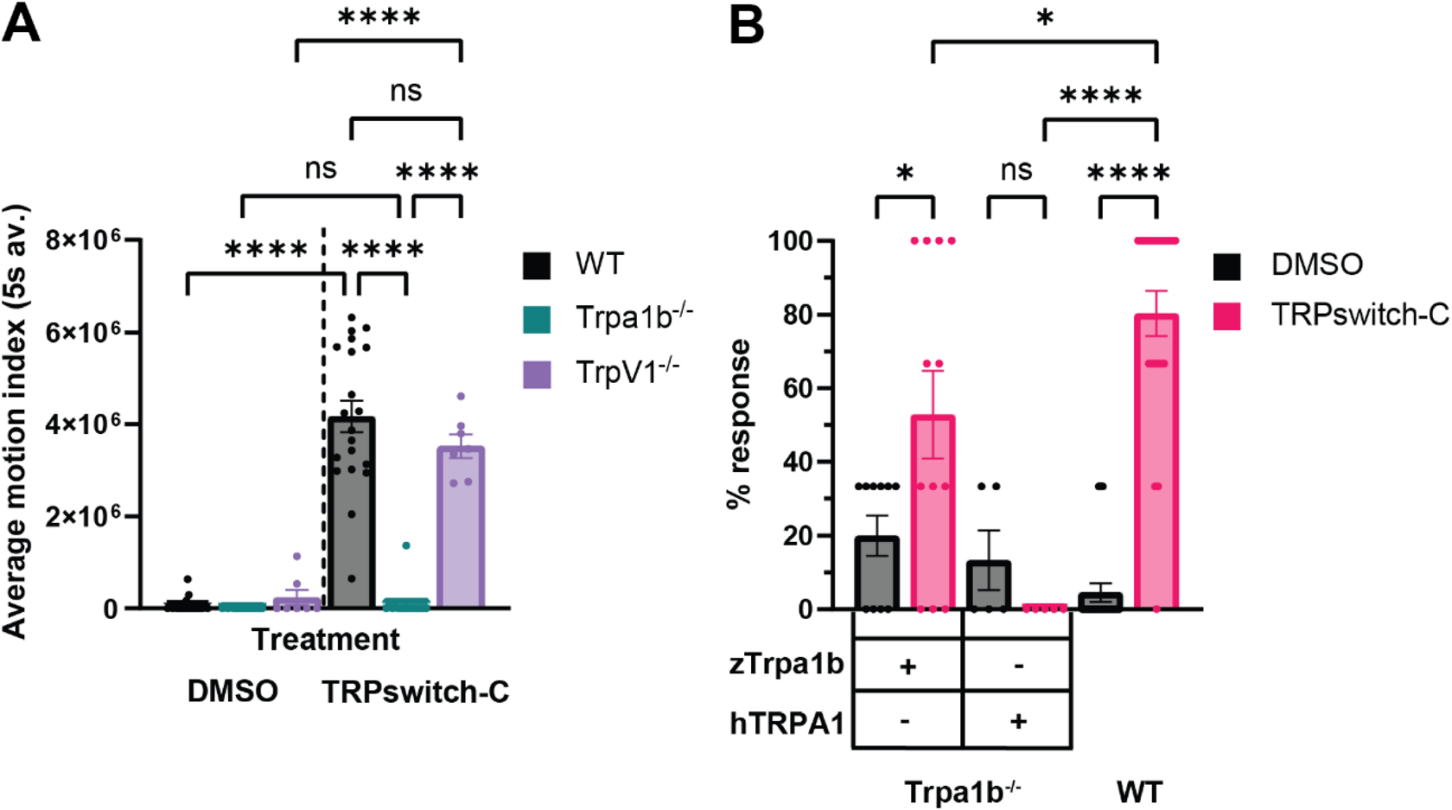
The activity of TRPswitch-C is specific for zebrafish Trpa1b. (A) Light induced motion response assay was performed with TRPswitch-C on WT, *trpa1b*^−/−^ mutant or *trpV1*^−/−^ mutant larvae. Values are mean ± SEM. (*p*-value **** ˂ 0.0001) see SI Movie 2. (B) Cross reactivity testing of TRPswitch-C on zebrafish Trpa1b ohnolog. Light induced motion response assays were performed on 2 dpf WT, *trpa1b*^−/−^ mutants or *trpa1b*^−/−^ mutants with transient mosaic expression of either zTrpa1b or hTRPA1 in Rohon Beard neurons. The percentage of larvae showing motion response upon light activation with either DMSO or 20 μM TRPswitch-C treatment was quantified. Each data point is a single larva. Values are mean ± SEM. (*p*-value ns ≥ 0.1234, * < 0.0332 or **** < 0.0001).

Two types of photoactivatable molecules have been previously reported that activate Trpa1b in zebrafish, Optovin,^20^ which is irreversible, and TRPswitch-A/B,^10^ which are reversible. In 3 dpf larval zebrafish, Trpa1b is normally expressed in Rohon–Beard neurons^21^ and its activation causes a characteristic motion response that is highly reproducible.^10,20,22^ A similar motion response was observed in zf larvae exposed to TRPswitch-C and light stimulation in comparison to the response of these prior compounds. To test whether Trpa1b was the target of TRPswitch-C, 3 dpf WT, *trpa1b*^−/−^*,* or *trpV1*^−/−^ mutant larvae were exposed to DMSO (1%) or TRPswitch-C (20 μM) and stimulated with 390 nm light. The motion response to TRPswitch-C was completely abolished in *trpa1b*^−/−^ mutant larvae (Fig. 4A and SI Movie 2). However, when *trpV1*^−/−^ mutants were exposed to TRPswitch-C, these larvae exhibited a similar motion response as WT larvae (Fig. 4A). These results suggest that the zTrpa1b channel is required for TRPswitch-C light-induced motion response.

To test the cross-reactivity of TRPswitch-C with other Trpa1 orthologs, we transiently expressed zebrafish Trpa1b (zTrpa1b) or human TRPA1 (hTRPA1) in Rohon Beard neurons of *trpa1b*^−/−^ mutant fish. Expression of zTrpa1b in *trpa1b*^−/−^ mutant fish restored the light induced motion response in fish exposed to TRPswitch-C upon 390 nm light activation (Fig. 4B). Conversely, expression of hTRPA1 in *trpa1b*^−/−^ mutant fish did not restore the light induced motion response in the presence of TRPswitch-C (Fig. 4B). These results suggest that the activity of TRPswitch-C is specific to the zebrafish Trpa1b and does not react with the human TRPA1 ortholog. This is also consistent with our previously reported compounds TRPswitch-A/B, despite the significantly different chemotype of TRPswitch-C. Zebrafish Trap1b and human TRPA1 share 46% amino acid sequence identity. The differences in amino acid sequences, which are distributed across various domains of TRPA1, may account for the specificity of TRPswitches towards zTrpa1b.

**Fig. 5:**
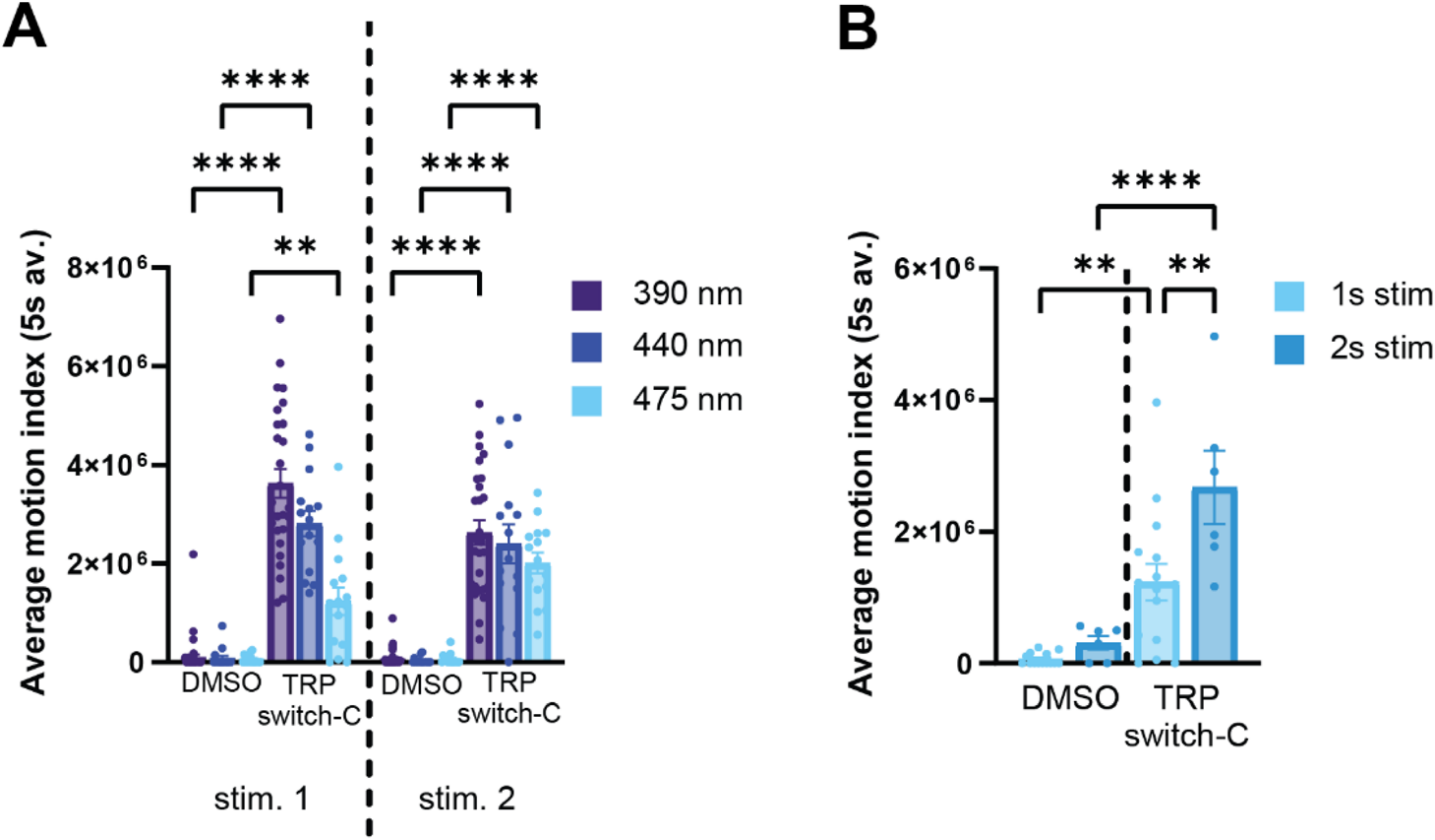
Comparing the light-induced activities of TRPswitch-C at different wavelengths. (A) Light-induced motion response assay was performed with TRPswitch-C on WT larvae at 390 nm, 440 nm or 475 nm. Values are mean ± SEM. (*p*-value ** < 0.0021; **** < 0.0001) see SI Movie 3. (B) Light-induced motion response assay was performed with TRPswitch-C on WT larvae at 475 nm for 1 s or 2 s stimulation. Values are mean ± SEM. (*p*-value ** < 0.0021; **** < 0.0001) see SI Movie 4.

Given the visible light addressability of aryl azopyrrole switches, we surveyed the light-induced motion response of TRPswitch-C at longer wavelengths. We found that TRPswitch-C can produce a significant light-induced motion response when stimulated with 440 nm and 475 nm light (Fig. 5A and SI Movie 3). No motion response was observed for 510 nm stimulation (data not shown), which likely relates to the lower absorbance and lower conversion to the active *Z* isomer at this wavelength (see Fig. 1). At longer wavelengths such as 475 nm the activity of TRPswitch-C increases with longer stimulation times (Fig. 5B), likely due to the lower absorbance at this wavelength. Together, this suggests that a range of wavelengths and irradiation times can be used for controlling the function of zTrpa1b when using TRPswitch-C.

**Fig. 6:**
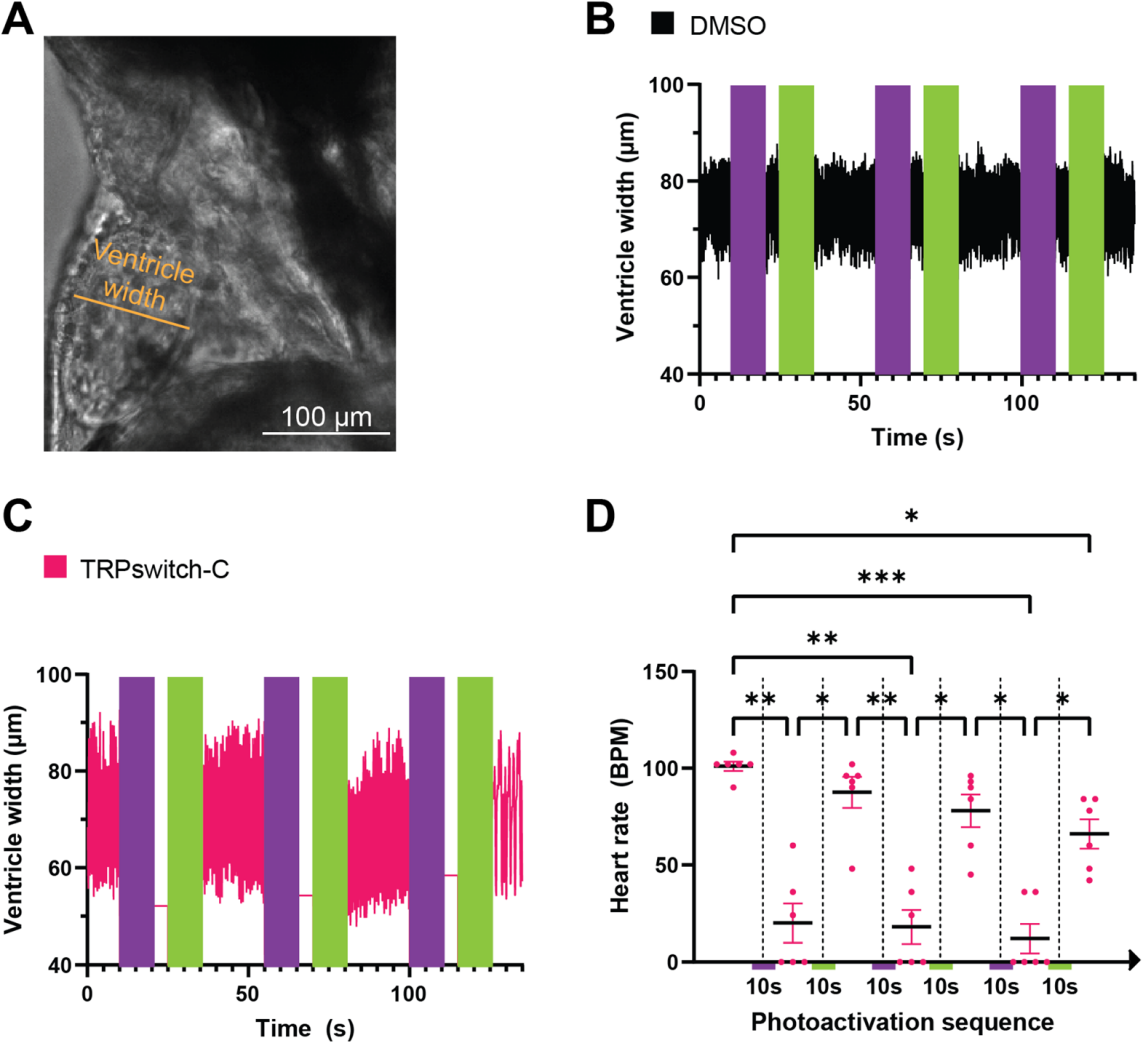
Photocontrol of larval zebrafish heartbeats using Trpa1b/TRPswitch-C. The heartbeat interruption assay was performed on *Tg(cmlc2:Trpa1b-2A-EGFP)* larvae in a *trpa1b*^−/−^ background. (A) Image of the zebrafish heart showing the ventricle chamber and the location of ventricle width measurements. (B and C) Representative traces of the ventricle width measured every 100 ms during the photoactivation sequence from larvae incubated in DMSO (B) or TRPswitch-C (C). Violet and green vertical lines indicate photoactivation with 10 s of 390 nm or 555 nm light, respectively. (D) Quantification of the heart rate (beat per minute, BPM) in larvae incubated in TRPswitch-C during the photoactivation sequence. Values are mean ± SEM. (*p*-value ns ≥ 0.1234; * < 0.0332; ** < 0.0021; *** < 0.0002). see SI Movie 5.

To demonstrate the reversible and repeatable activity of the Trpa1b/TRPswitch-C system, we utilized a previously established heartbeat interruption assay.^10^ This assay involves the use of 3 dpf zebrafish larvae from the transgenic line *Tg(cmlc2:Trpa1b-2A-EGFP)* in *trpa1b*^−/−^ mutants, in which Trpa1b is expressed exclusively in the cardiomyocyte of the larvae. Zebrafish larvae incubated in either DMSO or TRPswitch-C were then subjected to repeated cycles of activation and deactivation light. Since calcium is essential for heart contractions, the heartbeat serves as a biological readout of this assay—and by proxy, for the activation and deactivation of the Trpa1b channel (Fig. 6A). We found that when incubated in TRPswitch-C, activation with 390 nm light resulted in a stopping of the heartbeat, a response not observed in the DMSO controls (Fig. 6B and C). Subsequent illumination with deactivating 555 nm light was sufficient to restart the heartbeat (Fig. 6C and D). Importantly, repeated activation and deactivation cycles led to consistent stopping and restarting of the heartbeat (Fig. 6C and D), although with a progressive decrease in heart rate compared to the original baseline (Fig. 6D). Our results demonstrate that the Trpa1b channel can be repeatedly activated and deactivated with 390 nm and 555 nm light, respectively, in the presence of TRPswitch-C.

## Conclusions

In summary, we report the discovery of an aryl azopyrrole ligand, TRPSwitch-C, for the optical control of TRPA1, including in live zebrafish. The discovery of this ligand expands the tool molecules available to address TRPA1, which we expect to be particularly useful in optochemogenetic experiments studying excitable cells where a large depolarization current is needed. It seems that using the large absorption tuneability of heteroaromatic azos, together with suitable targets has large potential in future photopharmacology.

## Author contributions

Conceptualization: LCD, JAM, RTP, PYL, MJF; Funding acquisition: PJP, RTP, PYL, MJF; Investigation: LCD, JAM, KYZ, RXT, PRH; Methodology: LCD, JAM, RTP, PYL, MJF; Supervision: PJP, RTP, PYL, MJF; Writing – original draft: LCD, JAM, PYL, MJF; Writing – review & editing: all authors.

## Conflicts of interest

There are no conflicts to declare.

## Supplementary Material

SC-OLF-D5SC05070G-s001

SC-OLF-D5SC05070G-s002

SC-OLF-D5SC05070G-s003

SC-OLF-D5SC05070G-s004

SC-OLF-D5SC05070G-s005

SC-OLF-D5SC05070G-s006

## Data Availability

The data supporting this article have been included as part of the supplementary information (SI). Further raw data can be obtained from the authors upon request. Further experimental data is included in the SI and supplementary movie files. Supplementary information: SI Movie 1: Corresponding to Fig. 2C and D. Motion response of wild type zebrafish larvae in a 96 well plate treated with 1% DMSO (left) or 20 μM TRPswitch-C (right) in response to 1 s stimulation of 390 nm light illumination. Light illumination is indicated by the purple box in the corner of each well. SI Movie 2: Corresponding to Fig. 4A. Motion response of wild type (top row) or *trpa1b* mutant (bottom row) zebrafish larvae in a 96 well plate treated with 1% DMSO (left) or 20 μM TRPswitch-C (right) in response to 1 s stimulation of 390 nm light illumination. Light illumination is indicated by the purple box in the corner of each well. SI Movie 3: Corresponding to Fig. 5A. Motion response of wild type zebrafish larvae in a 96 well plate treated with 1% DMSO (top row) or 20 μM TRPswitch-C (bottom row) in response to 1 s stimulation of 390 nm (left), 440 nm (center), or 475 nm (right) light illumination. Light illumination is indicated by the corresponding-colored box between wells of the same stimulation condition. SI Movie 4: Corresponding to Fig. 5B. Motion response of wild type zebrafish larvae in a 96 well plate treated with 1% DMSO (top row) 20 μM TRPswitch-C (bottom row) in response to 1 s stimulation (left) or 2 s stimulation of 475 nm light illumination. Light illumination is indicated by the blue box in the corner of each well. SI Movie 5: Corresponding to Fig. 6. Heartbeat of *Tg(cmlc2:Trpa1b-2A-EGFP)* in a *trpa1b*^−/−^ background, shown in DMSO (left) or 20 μM TRPswitch-C (right) before and after the first stimulation cycle of the heartbeat interruption assay. Violet and green squares below the scale bar in each video indicate when 390 nm and 555 nm stimulations occur, respectively. The red circle indicates the area of stimulation. See DOI: https://doi.org/10.1039/d5sc05070g.
