# Supplementary material for: Aryl azopyrroles as visible light photoswitchable TRPA1 ligands
Source: Chem Sci. 2025 Sep 16;16(42):19777–85. doi: 10.1039/d5sc05070g (PMC12459677; doi:10.1039/d5sc05070g)
Supplement: SC-016-D5SC05070G-s001 [file SC-016-D5SC05070G-s001.pdf]

Supplementary Information (SI) for Chemical Science.
This journal is © The Royal Society of Chemistry 2025

Supplementary Information

Aryl azopyrroles as visible light photoswitchable TRPA1 ligands

Lisa C. Dollhopf[1,#], Jordan A. Munos[2,#], Kai Y. Zheng[3], Rui Xin Tao[1], Peter R. Haycock[1], Philip J. Parsons[1], Randall T. Peterson[4], Pui-Ying Lam[2,] and Matthew J. Fuchter[1,3,*]*

[1] Molecular Sciences Research Hub, Department of Chemistry, Imperial College London, Wood Lane, London W12 0BZ, United Kingdom

[2] Department of Cell Biology, Neurobiology and Anatomy, Medical College of Wisconsin, 53226 Milwaukee, WI, USA
*plam@mcw.edu

[3] Department of Chemistry, University of Oxford, Chemical Research Laboratory, 12 Mansfield Road, Oxford, OX1 3TA, United Kingdom
*matthew.fuchter@chem.ox.ac.uk

[4] Department of Pharmacology and Toxicology, University of Utah, Salt Lake City, UT, USA

General methods 2
Synthetic procedures and NMRs 3
Photochemistry 21
Animal Husbandry 25
Light induced motion response assay 25
Analysis 27
Heart experiments 27
References 29

General methods

All reagents and solvents were purchased from commercial vendors and used as received. Reactions were generally carried out under an inert atmosphere (N_2) and in anhydrous solvents with exception of the diazonium coupling which was conducted in an open flask behind a blast shield. All reactions were monitored via thin-layer chromatography (TLC) on Merck silica gel F254 plates (0.25 mm) that were visualized with UV light (254 nm) or one of the following TLC stains: ninhydrin for amines, dinitrophenylhydrazine (DNP) for aldehydes and ketones or $KMnO_4$ as a general stain.

NMR spectra were generally recorded in deuterated solvents ($CDCl_3$ or DMSO-d_6) using a 400 MHz Bruker AV NMR spectrometer, 500 MHz Bruker NMR spectrometer or 600 Hz Bruker Avance NEO NMR spectrometer (equipped with 5 mm helium cooled BBO cryoprobe) at 298K. Spectra were analyzed using MNova software (Version 14.3.1) and chemical shifts (δ) are reported in parts per million (ppm), referenced to the residual solvent peaks of the utilised deuterated solvent. Coupling constants (J) are reported in Hertz, using the following abbreviations for the multiplicity: s = singlet, d = doublet, t = triplet, q = quartet, m = multiplet and br = broad signal. ^{13}C NMR spectra were recorded with the equipment stated above at 101 MHz, 126 MHz or 151 MHz respectively as ^{19}F NMR spectra were recorded at 377 MHz, 471 MHz or 565 MHz.

High resolution mass spectrometry (HRMS) samples were measured with a Micromass Autospec Premier and Micromass LCT Premier spectrometer by the Imperial College London Department of Chemistry Mass Spectrometry Service or with a Waters BioAccord 2 LC-MS system (bench-top TOF with Acquity LC system in direct infusion (loop injection) mode at the Department of Chemistry, University of Oxford.

LCMS analysis was additionally conducted at the Centre for Medicines Discovery, University of Oxford. Instrument: Waters LCMS system (Waters 2767 sample manager, Waters SFO System Fluidics organizer, Waters 2545

binary gradient module, Waters 2998 PDA detector, Waters 2424 ELS detector, Waters ACQUITY QDa detector); Column: Kinetex 5 µM EVO C18 column (100 mm × 3.0 mm, 100 Å); eluent A: 88% water, 10% acetonitrile, and 2% of 0.5 M ammonium bicarbonate, eluent B: 18% water, 80% acetonitrile, and 2% of 0.5 M ammonium bicarbonate; gradient: 0-0.35 min 5% B, 0.35-1.35 min 5-95% B, 1.35-2.1 min 95% B, 2.1-2.2 min 95-5% B, 2.2-3 min 5% B; flow 2 ml/min; wavelength: 210 nm to 650 nm.

Synthetic procedures

General procedure A: Diazonium coupling

Aniline (1 eq., up to 5 mmol) is dissolved in 14 ml 1M HCl (MeOH can be added to aid solubility) and cooled to 0 °C. 1.2 eq. of $NaNO_2$ were dissolved in water (1 ml/mmol) and dropwise added to the dissolved aniline. The reaction was stirred for 30 min at 0 °C. Pyrrole coupling partners (1 eq.) were dissolved in 7 ml EtOH and 3 eq. of NaOAc were dissolved in 4 ml water. EtOH and water solutions were combined, cooled to 0 °C and added to the reaction mixture over 5 minutes. The reaction was allowed to stir for another hour at 0 °C. The reaction was extracted with EtOAc and brine, dried over $MgSO_4$, filtered and the solvents were evaporated to yield the crude product.

General procedure B: N-methylation

The substrate (up to 5 mM) was dissolved in 10-20 ml anhydrous THF and cooled to 0 °C. 2 eq of NaH (60% in mineral oil) were added portionwise until no more hydrogen formation could be observed. The reaction was allowed to stir for 30 min at 0 °C before 2.05 eq. of iodoalkane were added dropwise via syringe, followed by subsequent heating to 60 °C for an hour. As heating was discontinued and the reaction was cooled to room temperature, the reaction was quenched by slow and dropwise addition of water and extracted with DCM and brine. The organic phase was dried over $MgSO_4$, filtered and the solvents were evaporated to yield the crude product.

Supplementary Scheme 1: Synthesis of aryl pyrrole

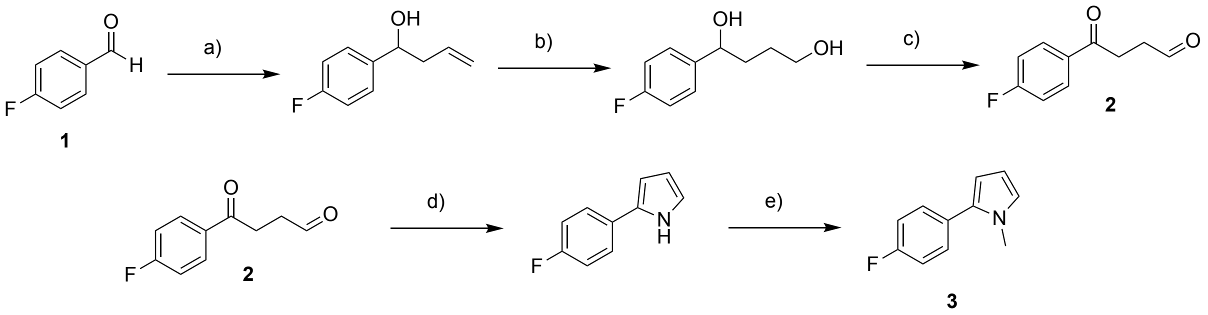

Reagents and conditions. a) Allyl magnesium chloride, THF, 0 °C to room temperature, 3h. b) 2M BH_3•DMS in THF, then aq. NaOH and H_2O_2, 0 °C to room temperature, 5h. c) $(COCl)_2$, DMSO, NEt_3, DCM, -78 °C to room temperature, 1.5 h, 33% over 3 steps. d) NH_4OAc, EtOH, reflux, 1.5 h, 85%. e) Iodomethane, NaH, THF, 0 °C to 60 °C, 1.5 h, 40-61%.

4-(4-fluorophenyl)-4-oxobutanal (2)

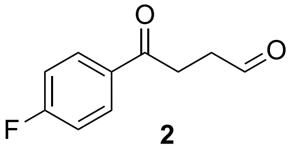

Ketone-aldehyde **2** was synthesised according to a known literature procedure from 4-fluorobenzaldehyde **1** in 3 steps.[1]

2-(4-fluorophenyl)-1*H*-pyrrole

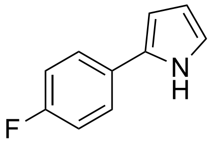

4-(4-Fluorophenyl)-4-oxobutanal (1.60 g, 8.90 mmol, 1 eq.) was dissolved in 30 ml EtOH, 8 eq. NH_4OAc were added to the solution and the reaction was heated to reflux for 1.5 h. The solvent was removed under reduced pressure and the crude was extracted with DCM and 5% $NaHCO_3$ solution. The organic phase was dried over $MgSO_4$, filtered, and the solvent was removed under vacuum to yield the desired product as a pink solid (1.22g, 85% yield).

^{1}**H NMR** (400 MHz, $CDCl_3$) δ 7.48 – 7.39 (m, 2H), 7.12 – 6.98 (m, 2H), 6.86 (td, *J* = 2.7, 1.4 Hz, 1H), 6.46 (ddd, *J* = 4.0, 2.7, 1.5 Hz, 1H), 6.30 (dt, *J* = 3.5, 2.6 Hz, 1H).

^{19}**F NMR** (377 MHz, $CDCl_3$) δ -116.29.

2-(4-fluorophenyl)-1-methyl-1*H*-pyrrole (3)

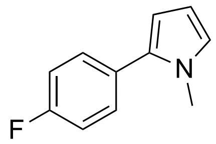

3

Compound **3** was synthesized according to General procedure B. 2-(4-fluorophenyl)-1*H*-pyrrole (1.20g, 7.44 mmol, 1 eq.) was reacted with 595 mg NaH (60% dispersion in mineral oil; 2 eq., 14.88 mmol) and 962 μl iodomethane (2.05 eq., 15.3 mmol) to yield the crude product. Flash column chromatography (0-100% EtOAc in hexane) yielded the desired purified product as a red oil (795 mg, 61% yield).

^{1}**H NMR** (400 MHz, $CDCl_3$) δ 7.35 (ddd, *J* = 8.7, 5.4, 2.7 Hz, 2H), 7.16 – 7.03 (m, 2H), 6.71 (t, *J* = 2.3 Hz, 1H), 6.23 – 6.16 (m, 2H), 3.63 (s, 3H).

^{19}**F NMR** (377 MHz, $CDCl_3$) δ -115.60.

^{13}**C NMR** (101 MHz, $CDCl_3$) δ 162.07 (d, *J* = 246.1 Hz), 133.69, 130.48 (d, *J* = 8.0 Hz), 129.59 (d, *J* = 3.3 Hz), 123.67, 115.44 (d, *J* = 21.4 Hz), 108.76, 107.88, 35.07.

These spectra are in accordance with previously reported literature.[2]

Supplementary Scheme 2: Photoswitch syntheses

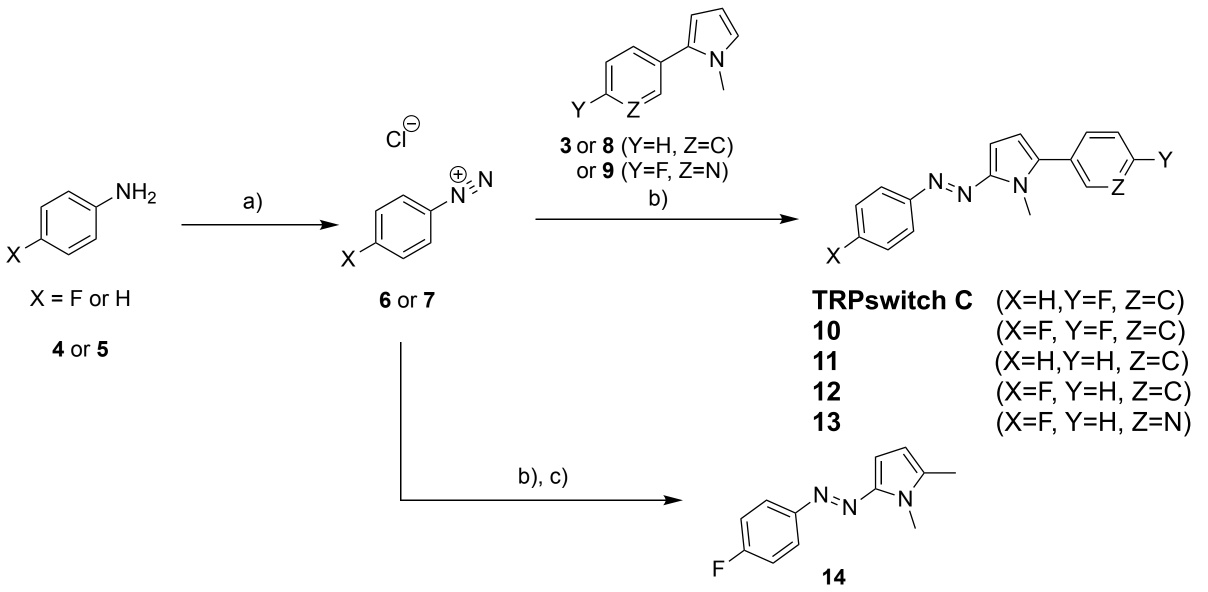

a) $NaNO_2$, 1M HCl, MeOH, 0 °C, 30 min; then b) pyrroles, NaOAc, EtOH, water, 0 °C, 1h, 8-28%. c) Iodomethane, NaH, THF, 0 °C to 60 °C, 1.5 h, 40-61%.

(*E*)-2-(4-fluorophenyl)-1-methyl-5-(phenyldiazenyl)-1*H*-pyrrole (TRPswitch C)

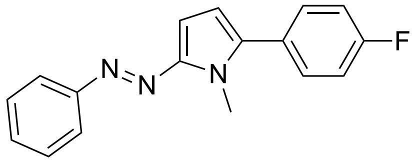

TRPswitch C

TRPswitch C was synthesised according to General procedure A. Aniline **5** (46 µl, 0.50 mmol, 1 eq.) was reacted with 2-(4-fluorophenyl)-1-methyl-1*H*-pyrrole **3** (88 mg, 0.50 mmol, 1 eq.) and purified by flash column chromatography (0-5% EtOAc in hexanes) to yield the desired product as an orange film (13 mg, 9% yield).

^{1}H NMR (400 MHz, $CDCl_3$) δ 7.85 (dq, *J* = 8.3, 1.2 Hz, 2H), 7.51 – 7.44 (m, 4H), 7.41 – 7.34 (m, 1H), 7.21 – 7.14 (m, 2H), 6.85 (d, *J* = 4.2 Hz, 1H), 6.40 (d, *J* = 4.2 Hz, 1H), 3.97 (s, 3H).

^{19}F NMR (377 MHz, $CDCl_3$) δ -113.42.

^{13}C NMR (101 MHz, $CDCl_3$) δ 162.64 (d, *J* = 248.2 Hz), 153.89, 147.82, 138.60, 130.66 (d, *J* = 8.1 Hz), 129.40, 129.11, 128.42 (d, *J* = 3.3 Hz), 122.17, 115.87 (d, *J* = 21.6 Hz), 111.50, 100.14, 31.63.

HRMS (ES+): *m/z* calculated 280.1250 for $C_{17}H_{15}N_3F$, found: 280.1248.

LCMS (3 min, pH8, ES+, 5-95% eluent B): Retention time of 2.11 min. Calc. purity: 96.21%.

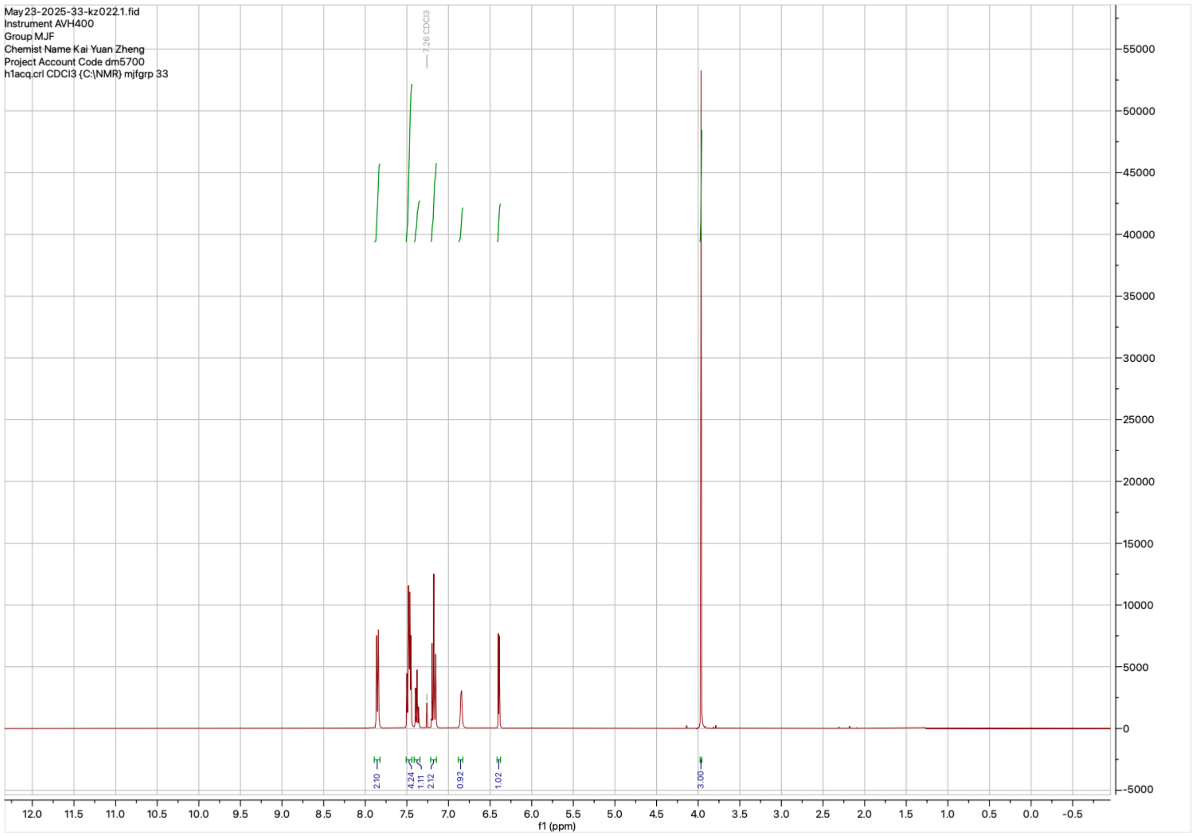

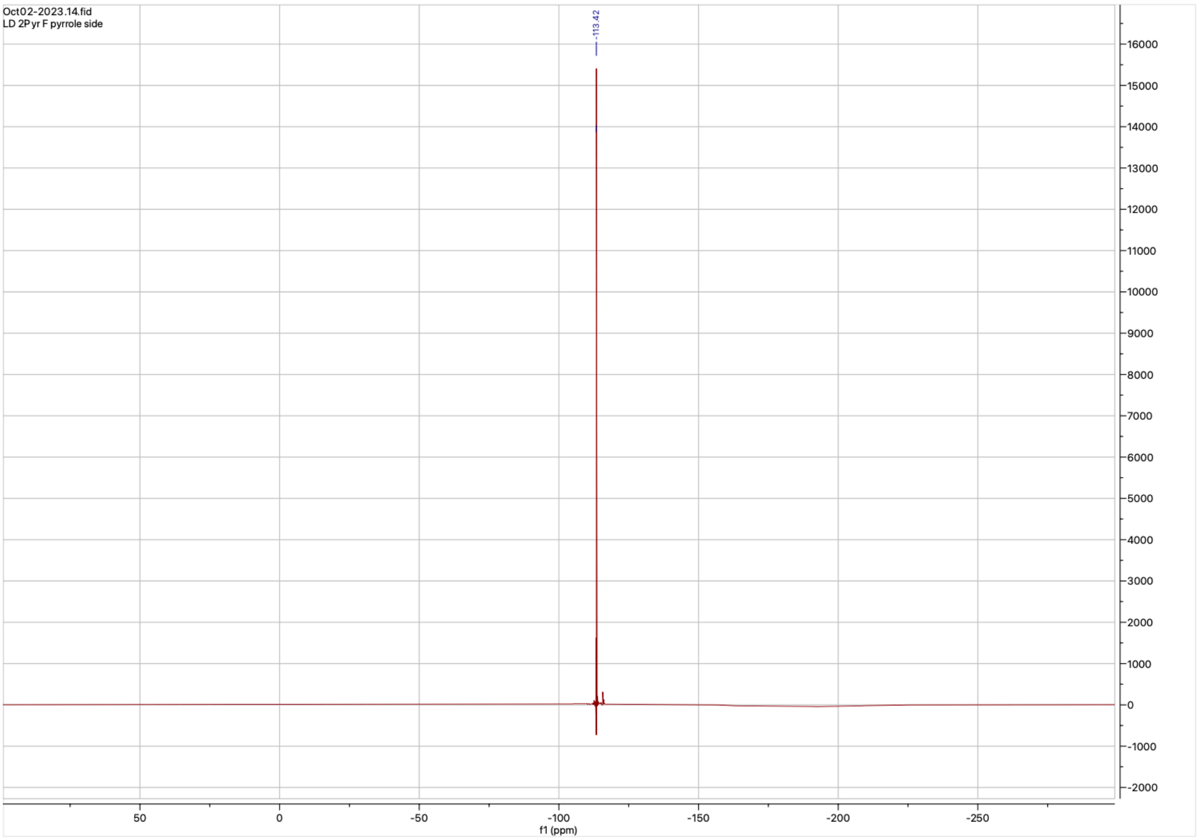
Oct02-2023.14.fid
LD 2Pyr F pyrrole side
-113.42
f1 (ppm)

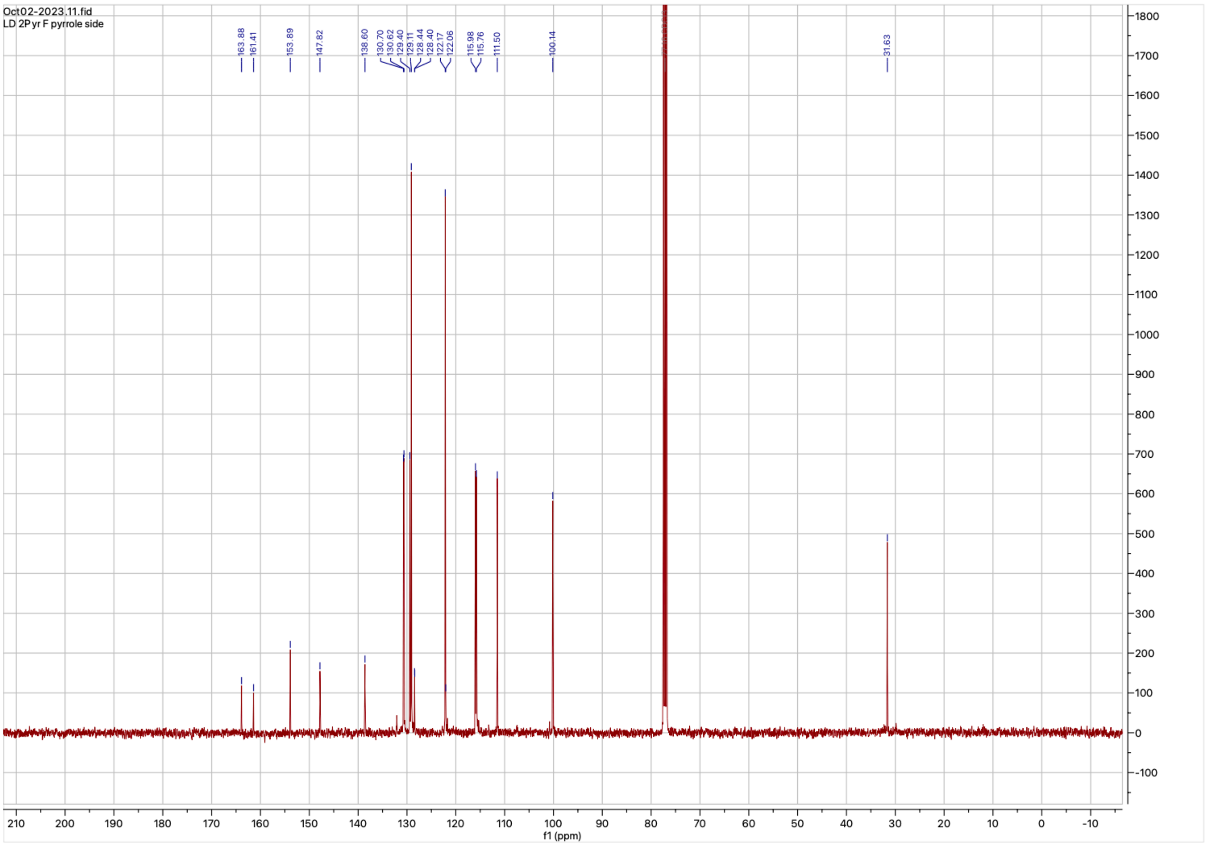
Oct02-2023.11.fid
LD 2Pyr F pyrrole side
163.88
161.41
153.89
147.82
138.60
130.70
130.62
129.40
129.11
128.44
128.40
122.17
122.06
115.98
115.76
111.50
100.14
31.63
f1 (ppm)

(E)-2-(4-fluorophenyl)-5-((4-fluorophenyl)diazenyl)-1-methyl-1H-pyrrole (10)

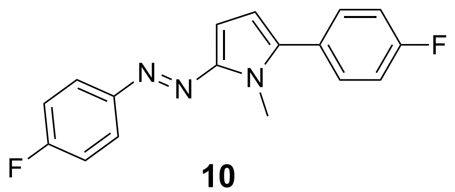

10

Compound **10** was synthesized according to General procedure A. 4-fluoroaniline **4** (110 μl, 1.13 mmol, 1 eq.) was reacted with 2-(4-fluorophenyl)-1-methyl-1H-pyrrole **9** (263 mg, 1.50 mmol, 1 eq.) and purified by flash column chromatography (100% hexane) to yield the desired product as an orange solid (36 mg, 8% yield).

^{1}H NMR (400 MHz, $CDCl_3$) δ 7.86 – 7.80 (m, 2H), 7.50 – 7.43 (m, 2H), 7.21 – 7.12 (m, 4H), 6.82 (s, 1H), 6.38 (d, J = 4.3 Hz, 1H), 3.95 (s, 3H).

^{19}F NMR (565 MHz, $CDCl_3$) δ -111.93, -112.75 – -113.75 (m).

^{13}C NMR (151 MHz, $CDCl_3$) δ 163.26 (d, J = 250.0 Hz), 162.62 (d, J = 248.3 Hz), 149.93, 147.40, 138.87,130.57 (d, J = 8.3 Hz), 128.13, 123.62 (d, J = 8.5 Hz), 116.14 – 115.57 (m), 111.70, 100.42, 31.52.

HRMS (ESI+): m/z calculated 298.1150 for $C_{17}H_{14}N_3F_2$, found: 298.1136.

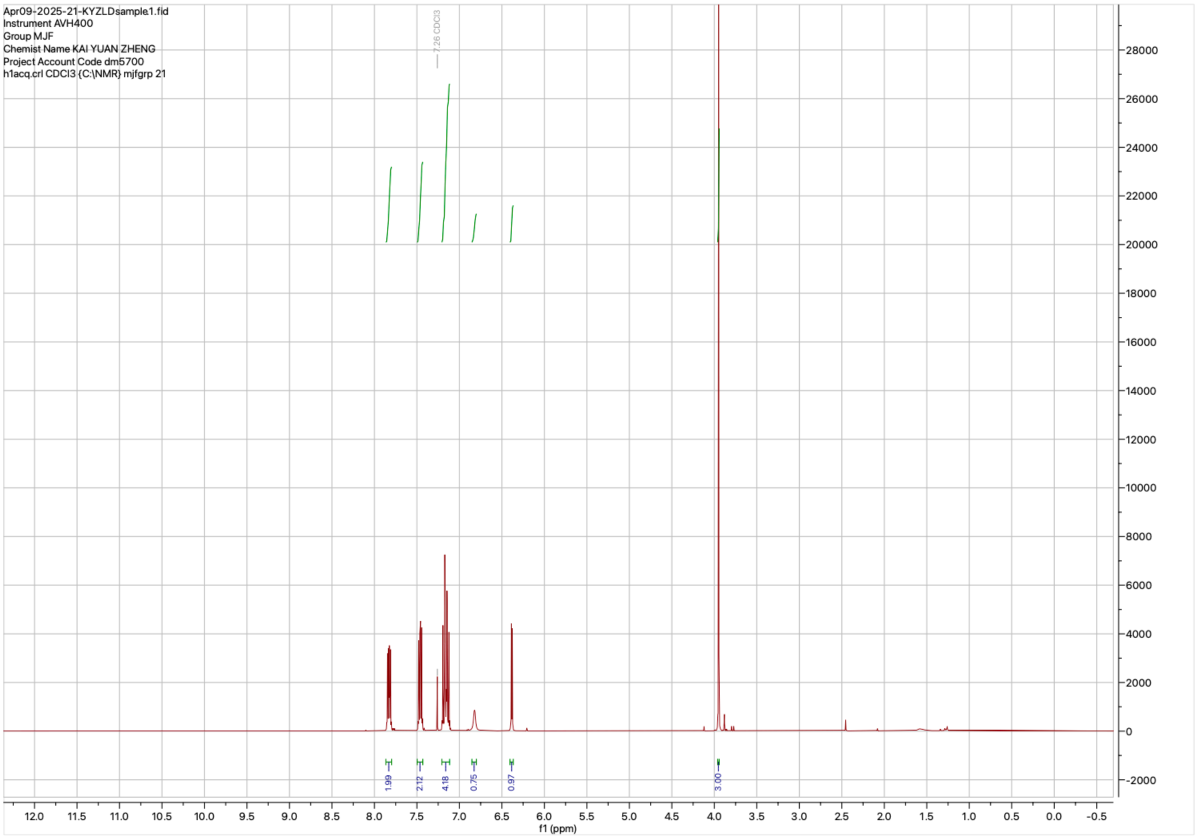
Apr09-2025-21-KYZLDsample1.fid
Instrument AVH400
Group MJF
Chemist Name KAI YUAN ZHENG
Project Account Code dm5700
h1acq.crl CDCl3 {C:\NMR} mjfgrp 21
7.26 CDCl3
1.99
2.12
4.18
0.75
0.97
3.00
f1 (ppm)

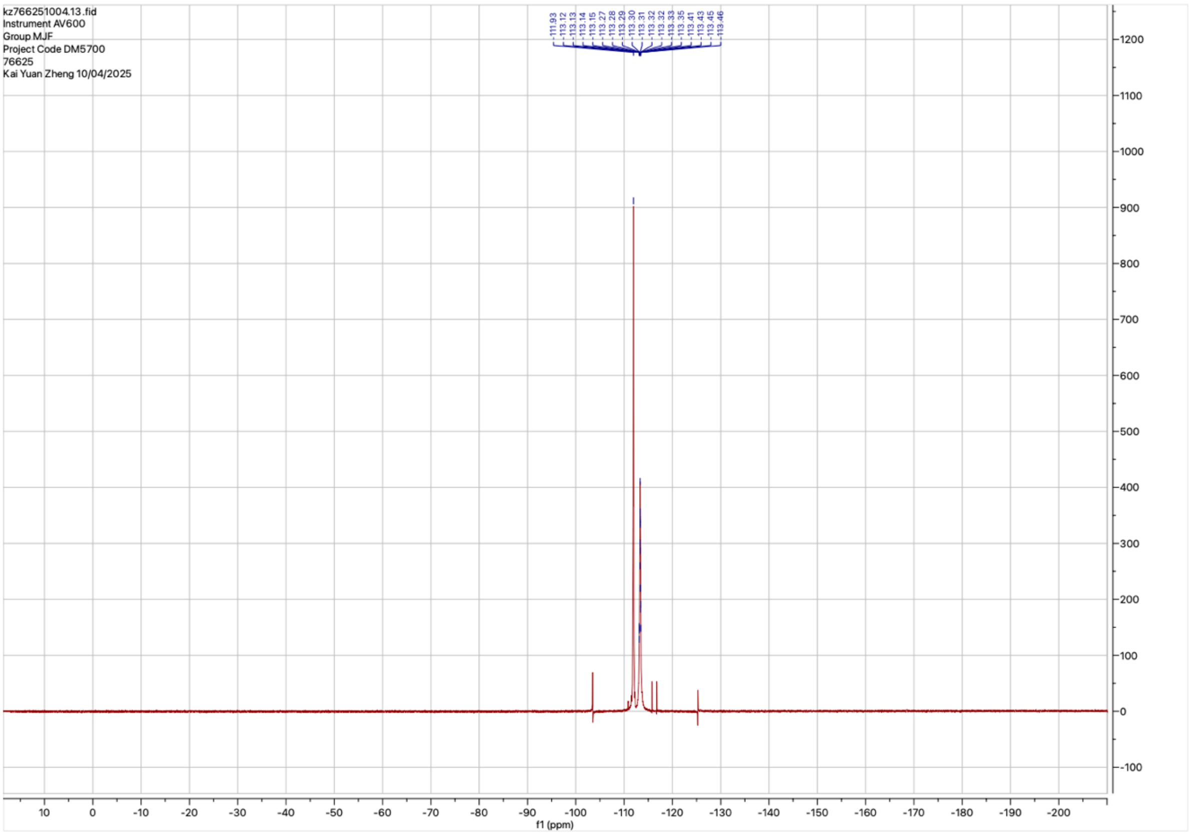
kz766251004.13.fid
Instrument AV600
Group MJF
Project Code DM5700
76625
Kai Yuan Zheng 10/04/2025
f1 (ppm)

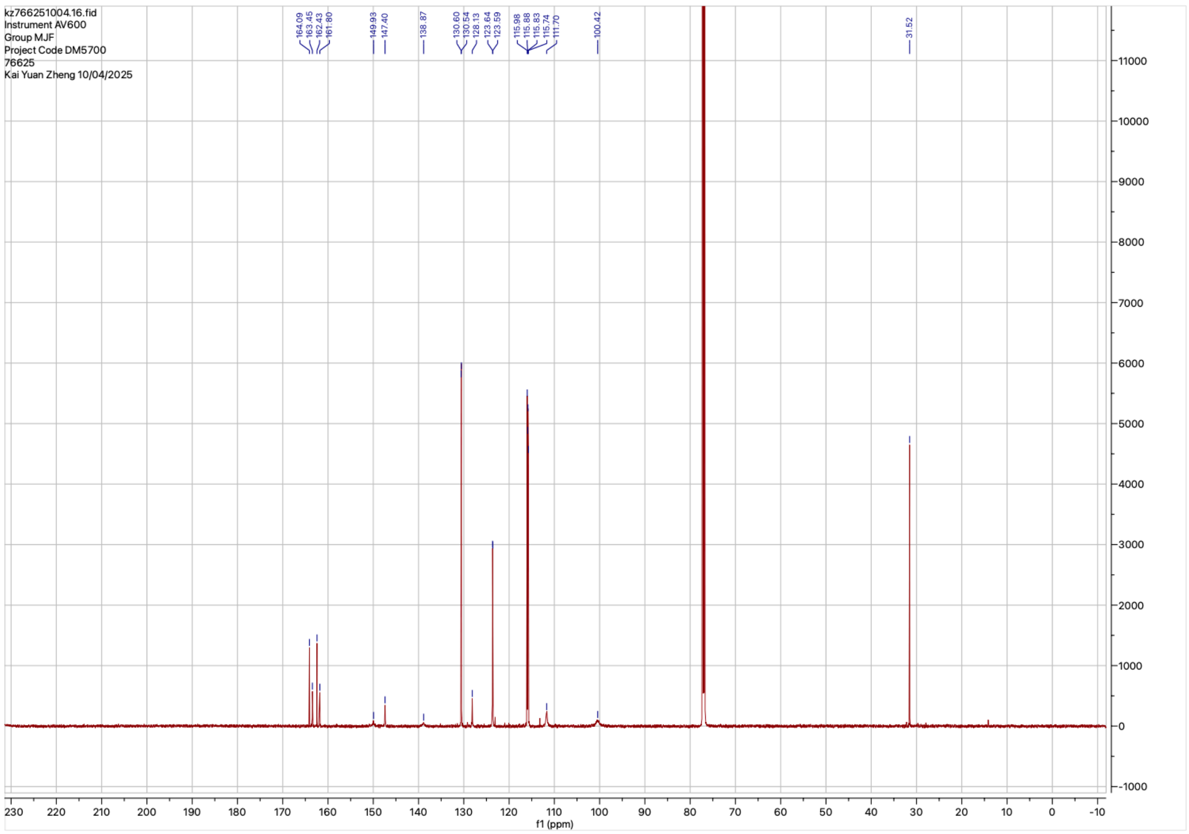

(*E*)-1-methyl-2-phenyl-5-(phenyldiazenyl)-1*H*-pyrrole (11)

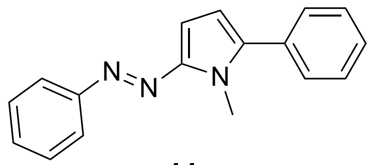

11

Compound **11** was synthesised according to General procedure A. Aniline **5** (44 μl, 0.48 mmol, 1 eq.) was reacted with 1-methyl-2-phenyl-1*H*-pyrrole **8** (88 mg, 0.48 mmol, 1 eq.) and purified by flash column chromatography (100% hexane) to yield the desired product as an orange film (20 mg, 16% yield).

^{1}H NMR (400 MHz, $CDCl_3$) δ 7.87 – 7.77 (m, 2H), 7.52 – 7.45 (m, 6H), 7.42 – 7.34 (m, 2H), 6.84 (d, *J* = 4.3 Hz, 1H), 6.43 (d, *J* = 4.3 Hz, 1H), 4.00 (s, 3H).

Note: ^{1}H NMR spectrum contains a peak at 1.60 that corresponds to residual water from the utilised deuterated chloroform.

^{13}C NMR (101 MHz, $CDCl_3$) δ 153.84, 147.79, 139.66, 132.17, 129.19, 128.98, 128.76, 128.67, 127.93, 122.04, 111.51, 100.11, 31.63.

HRMS (ES+): *m/z* calculated 262.1344 for $C_{17}H_{16}N_3$, found: 262.1340.

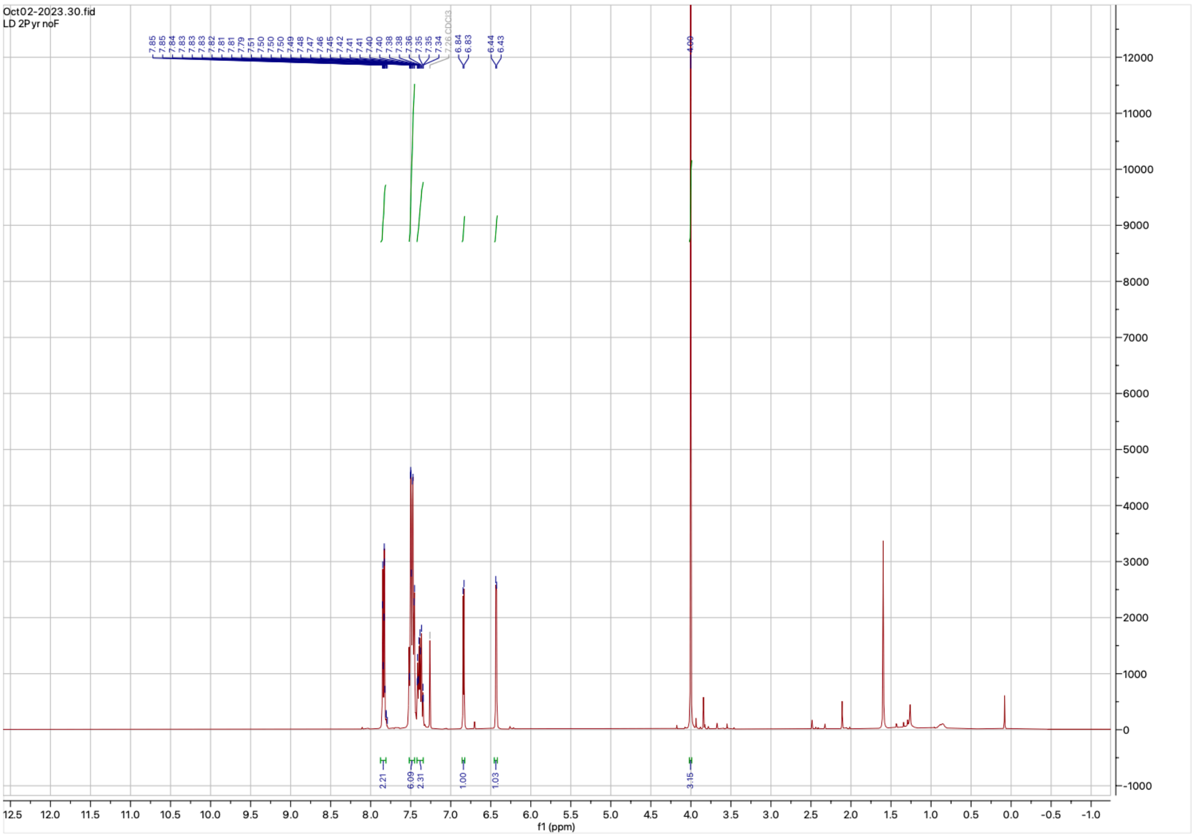

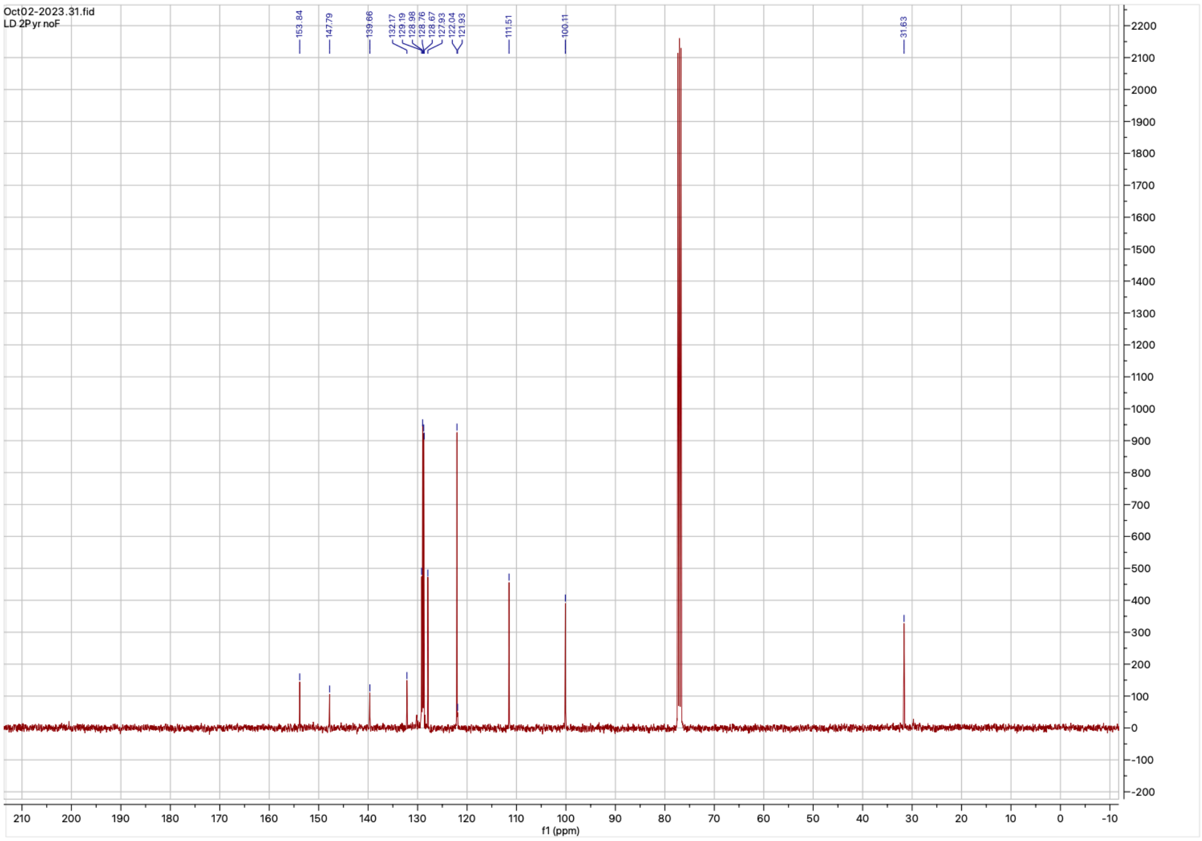

(*E*)-2-((4-fluorophenyl)diazenyl)-1-methyl-5-phenyl-1*H*-pyrrole (12)

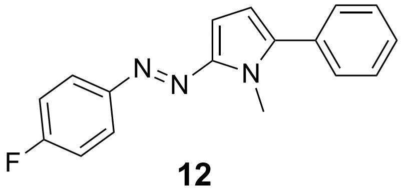

12

Compound **12** was synthesized according to General procedure A. 4-fluoroaniline **4** (45 μl, 0.48 mmol, 1 eq.) was reacted with 1-methyl-2-phenyl-1*H*-pyrrole **8** (88 mg, 0.48 mmol, 1 eq.) and purified by flash column chromatography (0-5% EtOAc in hexane) to yield the desired product as an orange film (16 mg, 12% yield).

^{1}H NMR (400 MHz, $CDCl_3$) δ 7.88 – 7.77 (m, 2H), 7.49 (qd, *J* = 8.2, 1.8 Hz, 4H), 7.42 – 7.36 (m, 1H), 7.20 – 7.09 (m, 2H), 6.82 (d, *J* = 4.3 Hz, 1H), 6.43 (d, *J* = 4.3 Hz, 1H), 3.99 (s, 3H).

Note: ^{1}H NMR spectrum contains a peak at 1.60 that corresponds to residual water from the utilised deuterated chloroform.

^{19}F NMR (377 MHz, $CDCl_3$) δ -112.16.

^{13}C NMR (101 MHz, $CDCl_3$) δ 163.33 (d, *J* = 249.7 Hz), 150.48 (d, *J* = 3.0 Hz), 147.74, 139.81, 132.23, 128.96 – 128.64 (m), 128.08, 123.78 (d, *J* = 8.5 Hz), 115.98 (d, *J* = 22.9 Hz), 111.64, 100.24, 31.73.

HRMS (ES+): *m/z* calculated 280.1250 for $C_{17}H_{15}N_3F$, found: 280.1258

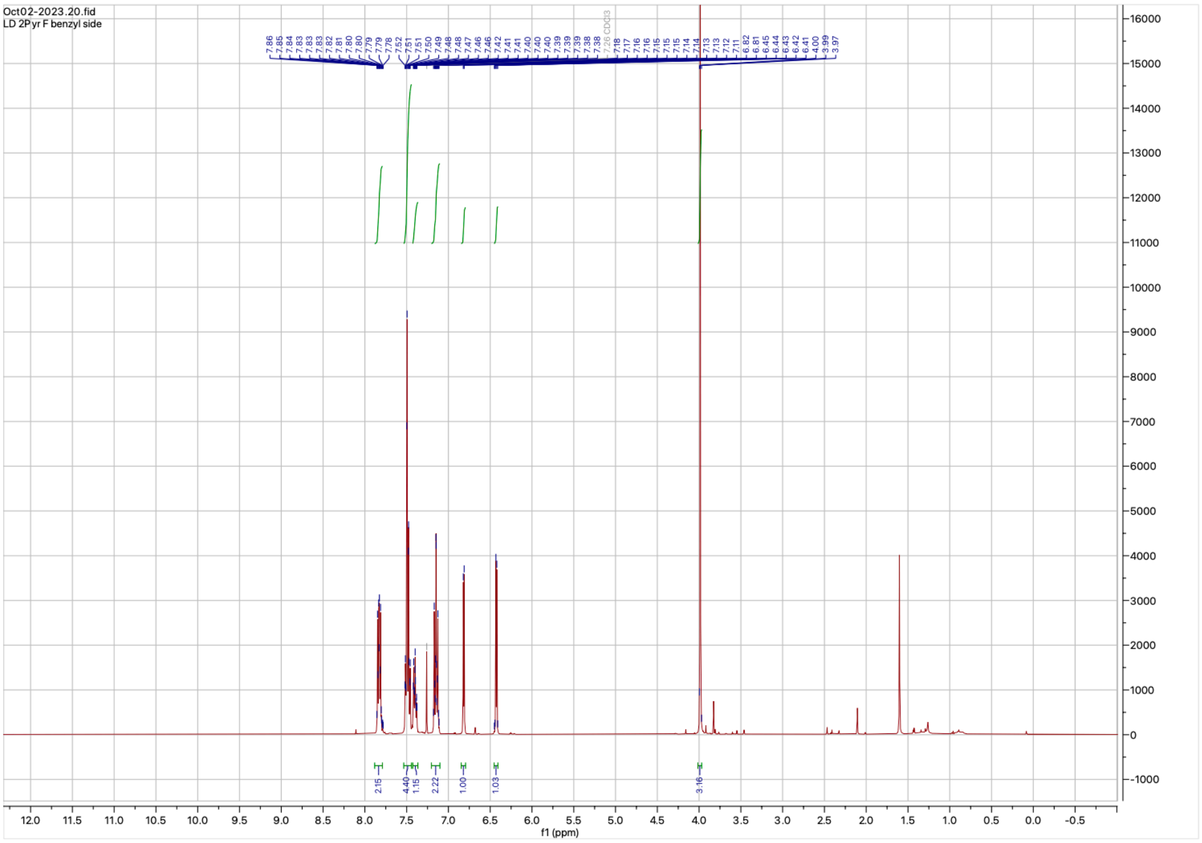

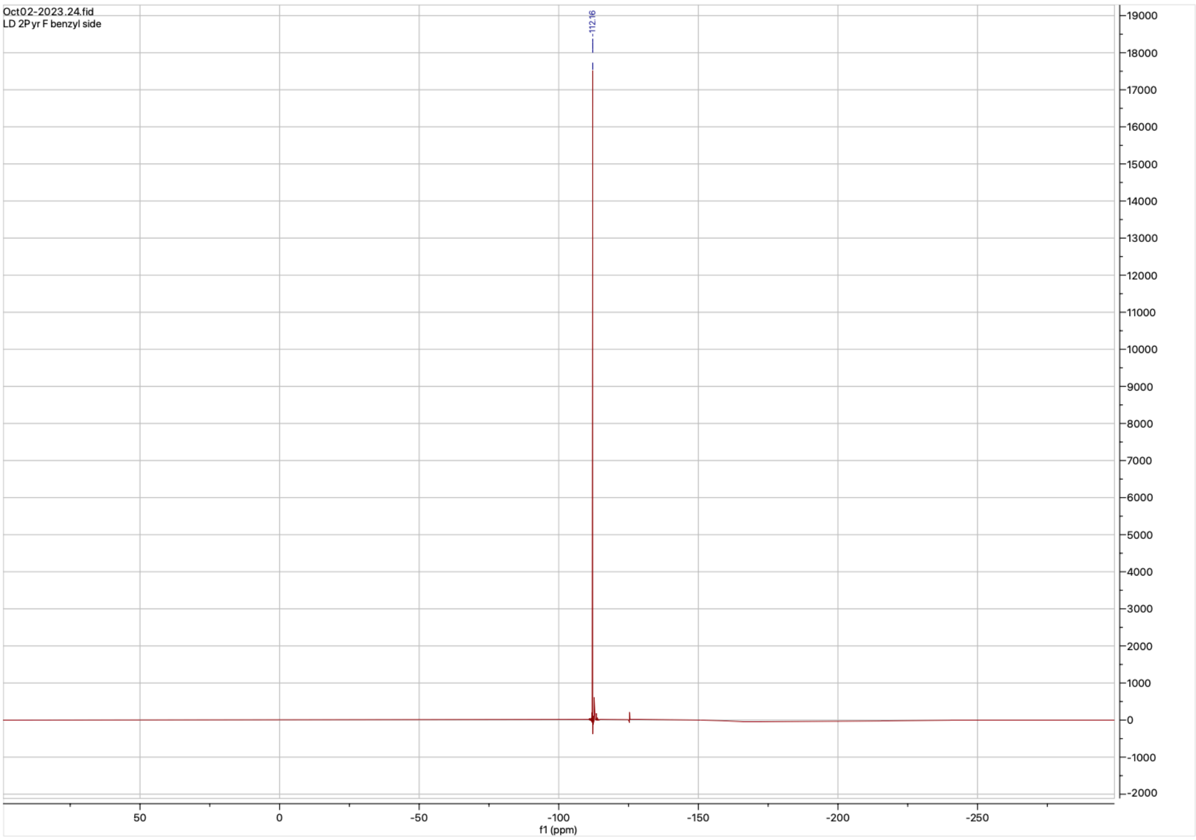
Oct02-2023.24.fid
LD 2Pyr F benzyl side
-112.16
f1 (ppm)

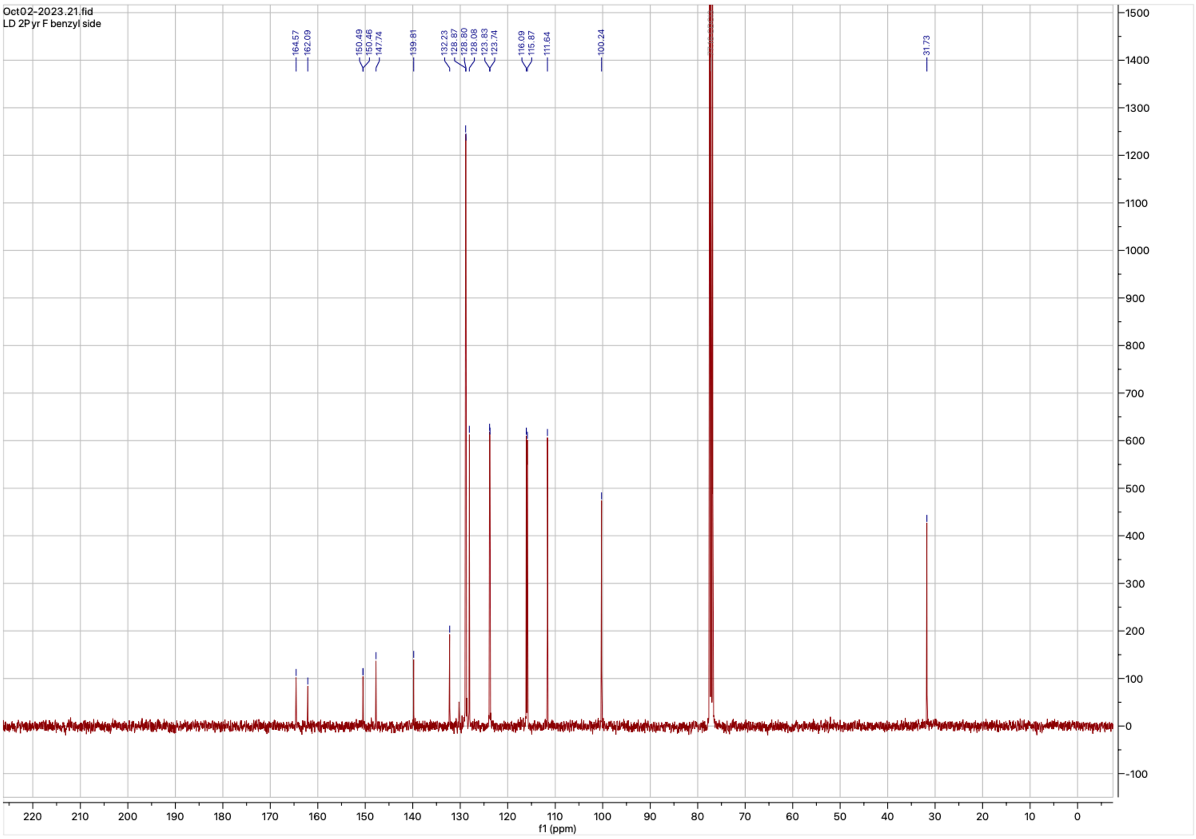
Oct02-2023.21.fid
LD 2Pyr F benzyl side
164.57
162.09
150.49
150.46
147.74
139.81
132.23
128.87
128.80
128.08
123.83
123.74
116.09
115.87
111.64
100.24
31.73
f1 (ppm)

(*E*)-3-(5-((4-fluorophenyl)diazenyl)-1-methyl-1*H*-pyrrol-2-yl)pyridine (13)

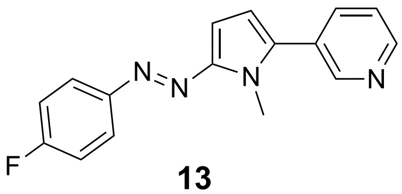

13

Compound **13** was synthesized according to General procedure A. 4-fluoroaniline **4** (60 μl, 0.63 mmol, 1 eq.) was reacted with nicotyrine **9** (100 mg, 0.63 mmol, 1 eq.) and purified by flash column chromatography (0-20% EtOAc in hexane) to yield the desired product as an orange solid (50 mg, 28% yield).

^{1}H NMR (600 MHz, $CDCl_3$) δ 8.81 – 8.79 (m, 1H), 8.63 (dd, *J* = 4.9, 1.6 Hz, 1H), 7.89 – 7.82 (m, 3H), 7.46 (ddd, *J* = 7.9, 4.9, 0.9 Hz, 1H), 7.19 – 7.13 (m, 2H), 6.82 (d, *J* = 4.3 Hz, 1H), 6.49 (d, *J* = 4.3 Hz, 1H), 4.01 (s, 3H).

^{19}F NMR (565 MHz, $CDCl_3$) δ -111.22.

^{13}C NMR (151 MHz, $CDCl_3$) δ 163.68 (d, *J* = 250.6 Hz), 150.27 (d, *J* = 3.1 Hz), 148.63, 148.36, 148.04, 136.47, 135.18, 128.78, 124.06 (d, *J* = 8.5 Hz), 123.88, 116.09 (d, *J* = 22.9 Hz), 112.37, 100.32, 31.76.

HRMS (ESI+): *m/z* calculated 281.1197 for $C_{16}H_{14}N_4F$, found: 281.1207.

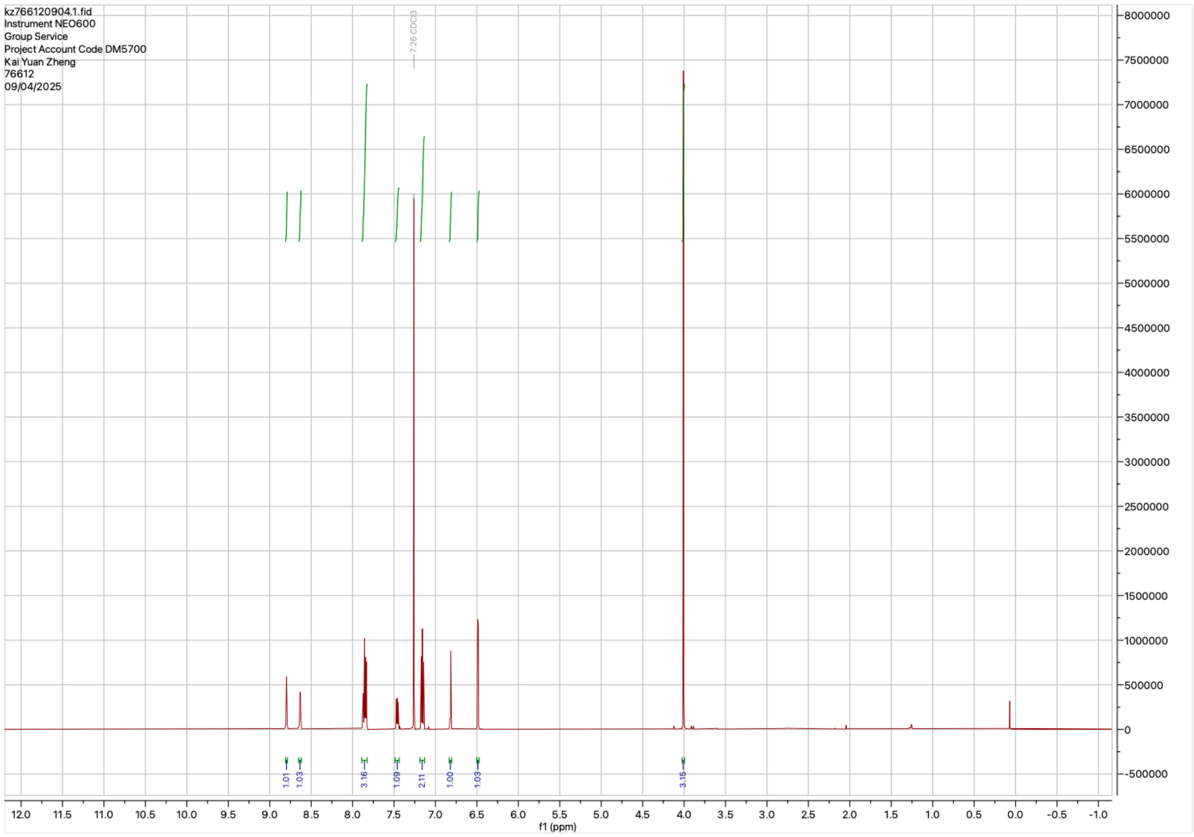
kz766120904.1.fid
Instrument NEO600
Group Service
Project Account Code DM5700
Kai Yuan Zheng
76612
09/04/2025
7.26 CDCl3
1.01
1.03
3.16
1.09
2.11
1.00
1.03
3.15
f1 (ppm)

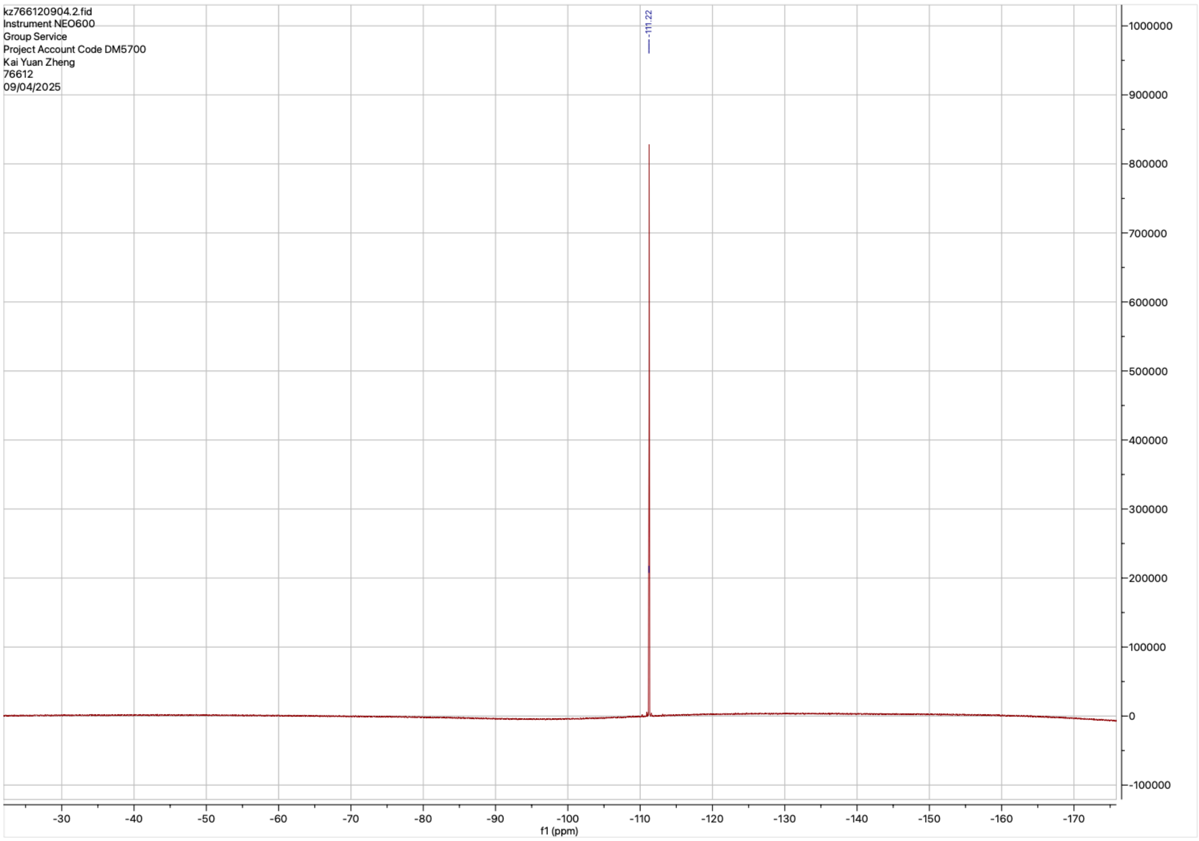
kz766120904.2.fid
Instrument NEO600
Group Service
Project Account Code DM5700
Kai Yuan Zheng
76612
09/04/2025
-111.22
f1 (ppm)

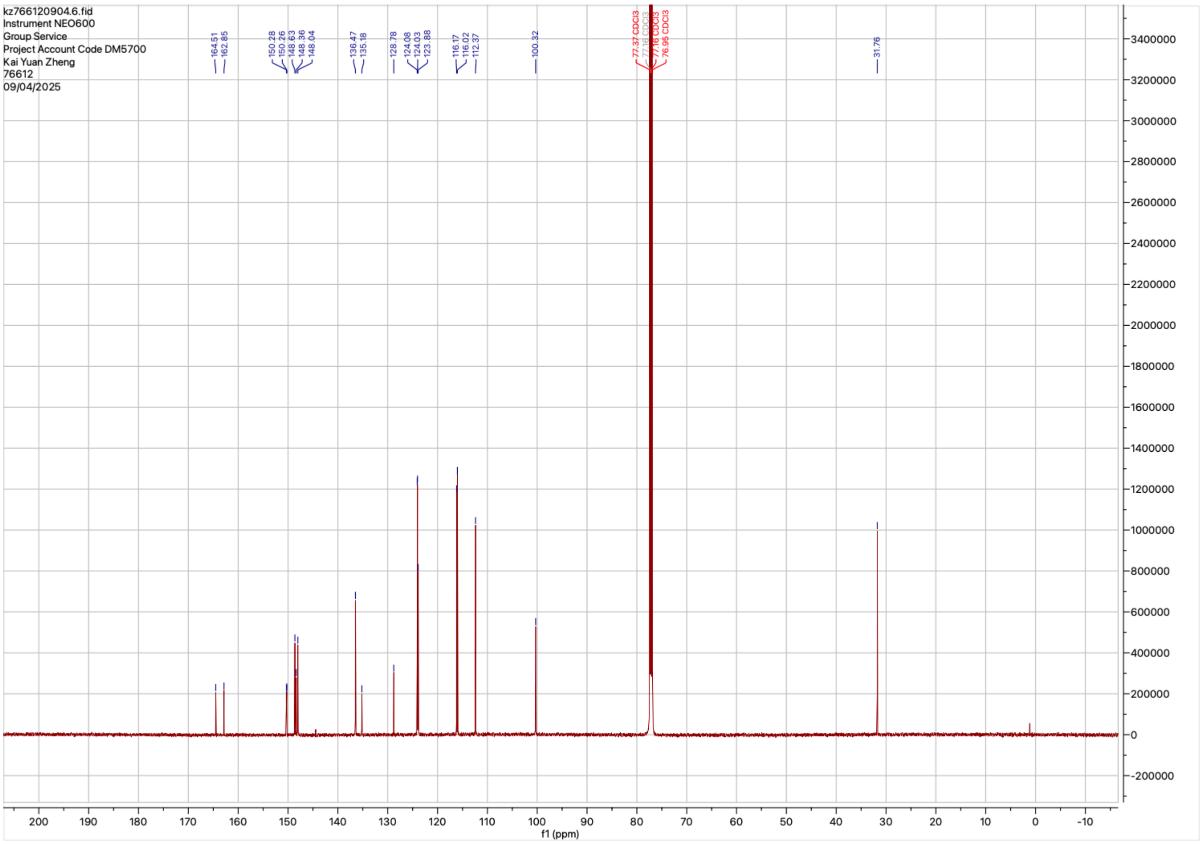

(*E*)-2-((4-fluorophenyl)diazenyl)-1,5-dimethyl-1*H*-pyrrole (14)

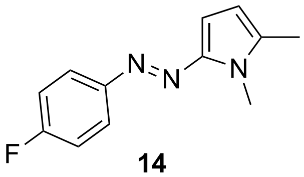

14

Compound **14** was synthesized according to General procedures A and B in 2 steps. 4-fluoroaniline 4 (235 μl, 2.47 mmol, 1 eq.) was reacted with 2-methyl-1*H*-pyrrole (207 μl, 2.47 mmol, 1 eq.) to yield the desired crude intermediate which was passed through a silica plug (100% hexane) to remove any polar impurities. As the second step, the intermediate (90 mg, 0.44 mmol, 1 eq.) was N-methylated using iodomethane (56 μl,0.90 mmol, 2.05 eq.) and purification by flash column chromatography (0-20% EtOAc in hexane) yielded the desired compound as a red-brown solid (100 mg, 19% yield).

^{1}**H NMR** (400 MHz, $CDCl_3$) δ 7.79 (dd, *J* = 8.3, 5.2 Hz, 2H), 7.17 – 7.08 (m, 2H), 6.74 (s, 1H), 6.12 (s, 1H), 3.84 (s, 3H), 2.36 (s, 3H).

^{19}**F NMR** (565 MHz, $CDCl_3$) δ -112.97.

^{13}**C NMR** (151 MHz, $CDCl_3$) δ 162.91 (d, *J* = 249.1 Hz), 150.00, 146.05, 135.72, 123.22, 115.81 (d, *J* = 22.9 Hz), 110.27, 99.51, 29.75, 12.87.

HRMS (ES+): *m/z* calculated 218.1088 for $C_{12}H_{13}N_3F$, found: 218.1078.

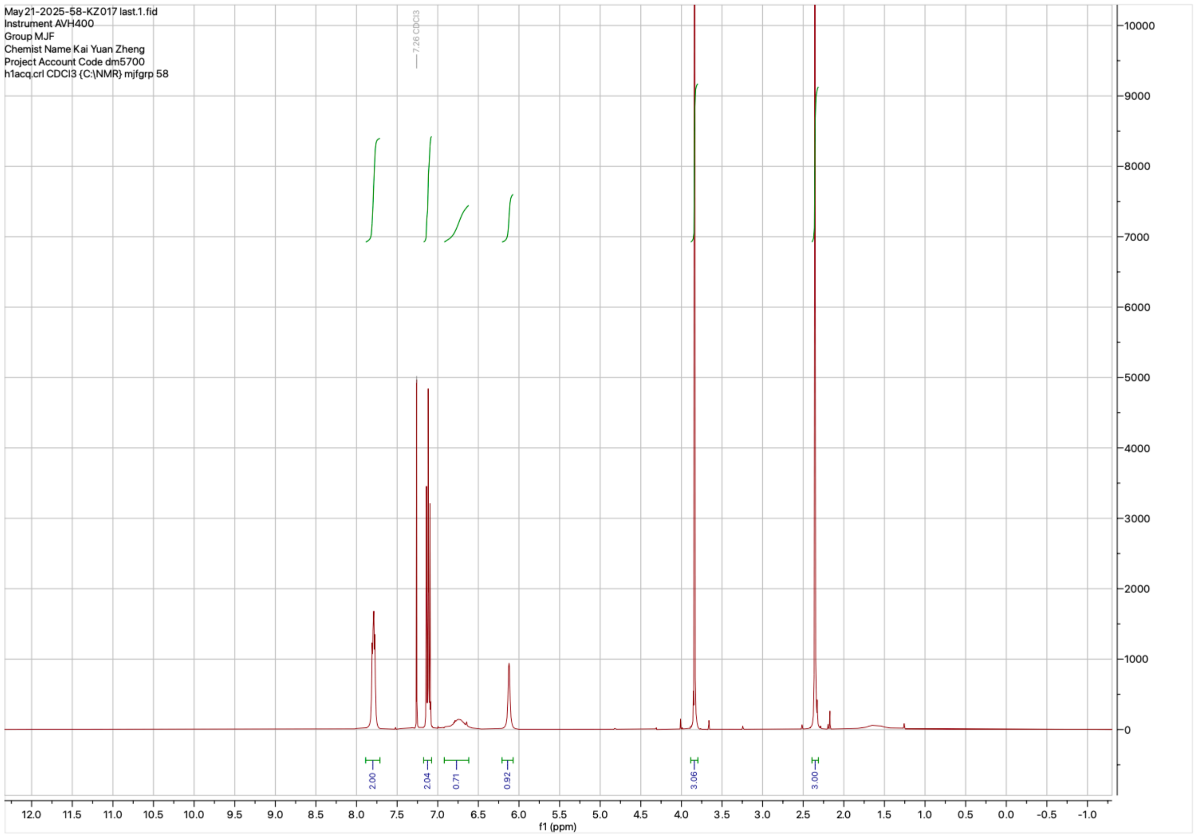

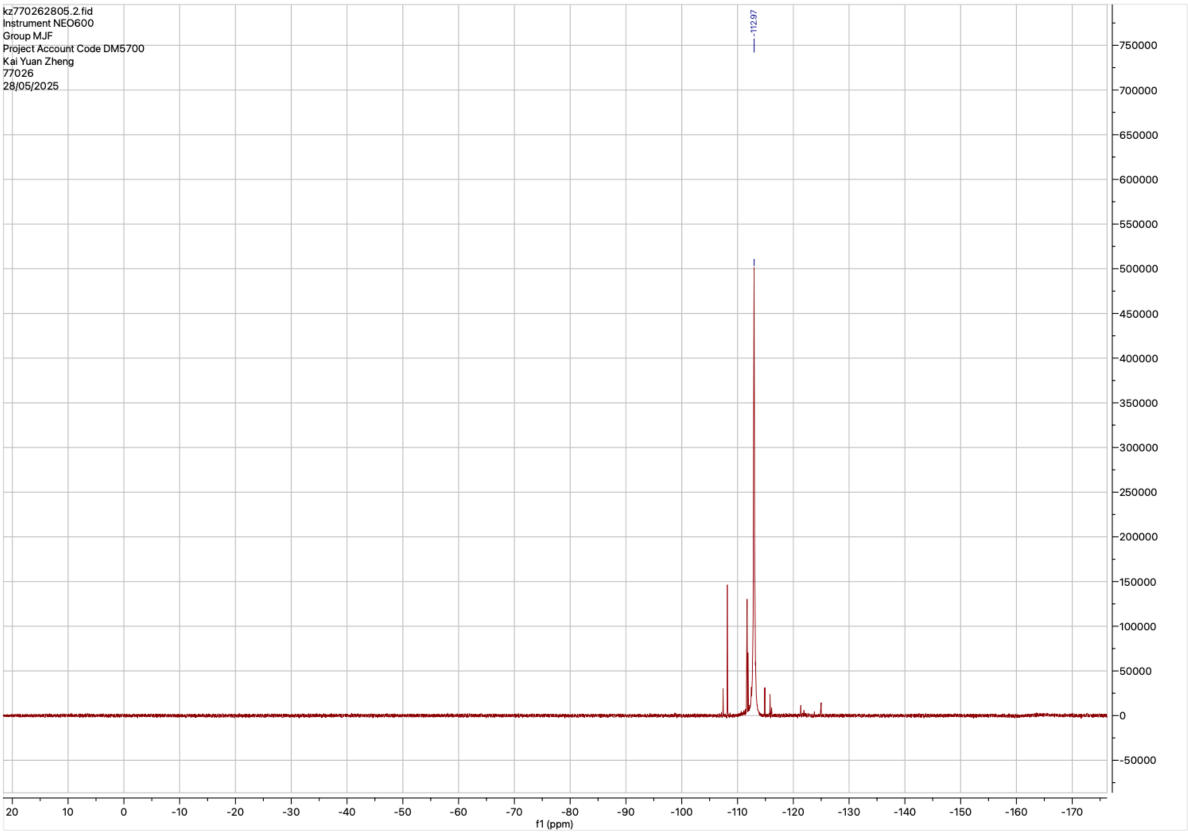
kz770262805.2.fid
Instrument NEO600
Group MJF
Project Account Code DM5700
Kai Yuan Zheng
77026
28/05/2025
-112.97
f1 (ppm)

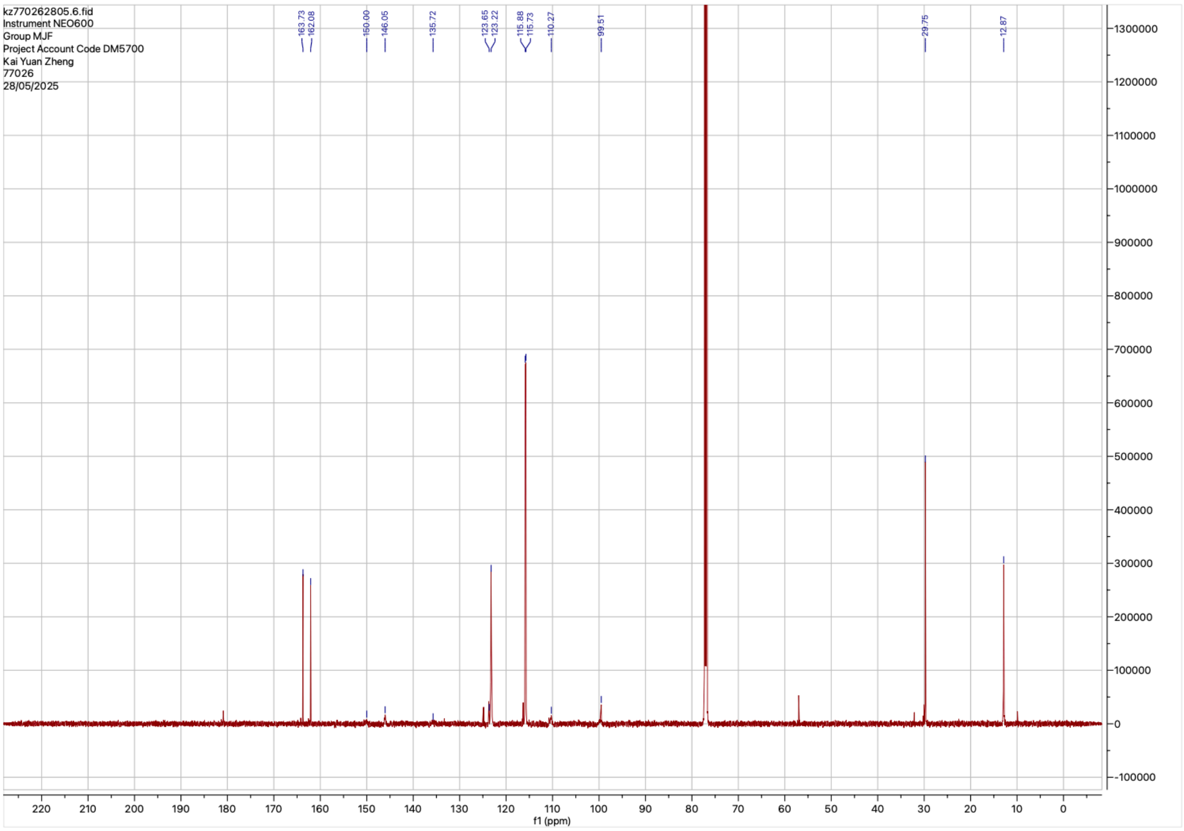
kz770262805.6.fid
Instrument NEO600
Group MJF
Project Account Code DM5700
Kai Yuan Zheng
77026
28/05/2025
163.73
162.08
150.00
146.05
135.72
123.65
123.22
115.88
115.73
110.27
99.51
29.75
12.87
f1 (ppm)

Photochemistry

Samples were kept in the dark and dark-adapted (heated and stirred in the dark for several hours) if required to obtain pure *E* isomer samples. For all samples, a 1 mM stock solution in solvent was prepared (generally from DMSO, HPLC grade and anhydrous) and said stock was diluted with solvent as needed. Samples that were prepared and stored in the dark were assumed to consist of 100% *E*-isomer.

Absorption spectra for UV-vis spectroscopy were recorded on an Agilent Cary 60 UV-vis spectrometer which was equipped with an external temperature probe and quartz cuvette with a screw top lid (1 cm pathlength) were used. Blank measurements of pure solvent were recorded and deducted from any following measurements. Diluted sample solutions were irradiated in the utilized cuvette using a custom setup from Sahlmann Photochemical Solutions, utilizing LEDs of several wavelengths: 340 nm (Seoul CUD4AF1B LEDs, 3x 50 mW, peak wavelength: 346 nm, FWHM: 9nm), 365 nm (Nichia NCSU276A LEDs, 3x 800 mW, peak wavelength: 368 nm, FWHM: 9nm), 405 nm (Nichia NCSU199C LEDs, 3x 770 mW, peak wavelength: 409 nm, FWHM: 11 nm), 450 nm (Nichia NCSC219B-V1 LEDs, 3x 900 mW, peak wavelength: 444 nm, FWHM: 18 nm), 495 nm (Nichia NCSE199B-V1 LEDs, 3x 750 mW, peak wavelength: 497 nm, FWHM: 28 nm), 525 nm (Nichia NCSG219B-V1 LEDs, 3x 450 mW, peak wavelength 520 nm, FWHM: 38 nm) or 630 nm (NCSR219B-V1 LEDs, 3x 780 mW, peak wavelength: 636 nm, FWHM: 16 nm). All LEDs were used at 100% power and samples were generally irradiated for 3 minutes until the respective photostationary state was reached.

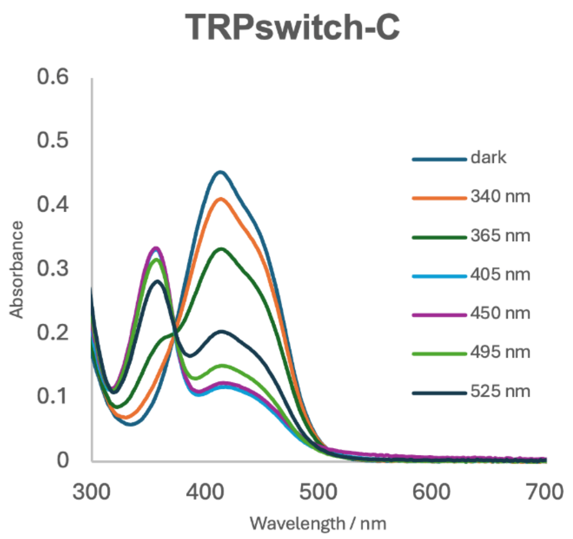
TRPswitch-C
Absorbance
Wavelength / nm
0
0.1
0.2
0.3
0.4
0.5
0.6
300
400
500
600
700
dark
340 nm
365 nm
405 nm
450 nm
495 nm
525 nm

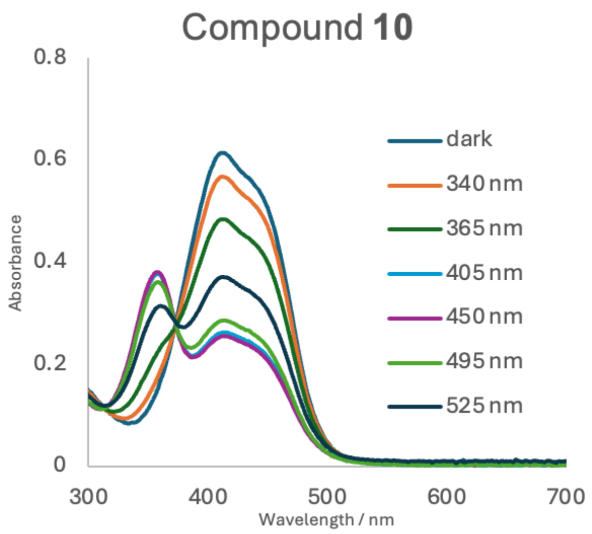
Compound 10
Absorbance
Wavelength / nm
0
0.2
0.4
0.6
0.8
300
400
500
600
700
dark
340 nm
365 nm
405 nm
450 nm
495 nm
525 nm

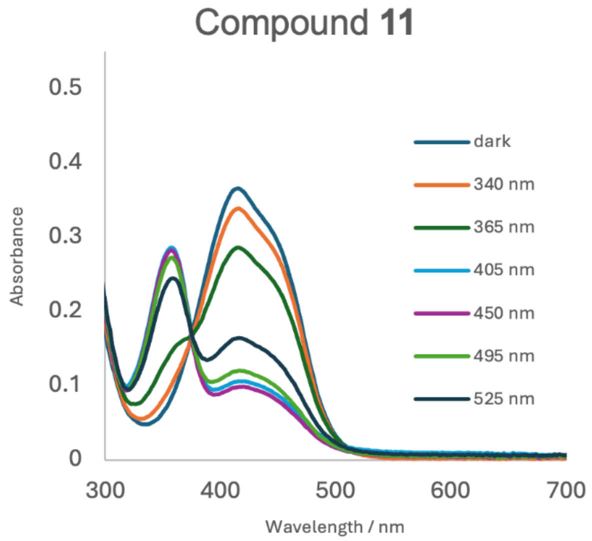
Compound 11
Absorbance
Wavelength / nm
0
0.1
0.2
0.3
0.4
0.5
300
400
500
600
700
dark
340 nm
365 nm
405 nm
450 nm
495 nm
525 nm

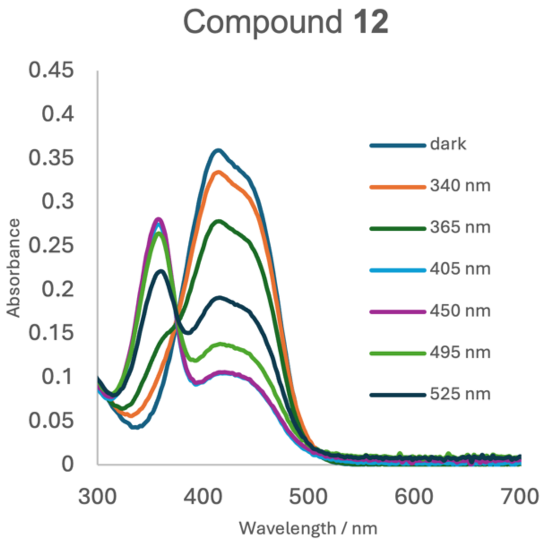
Compound 12
Absorbance
Wavelength / nm
0
0.05
0.1
0.15
0.2
0.25
0.3
0.35
0.4
0.45
300
400
500
600
700
dark
340 nm
365 nm
405 nm
450 nm
495 nm
525 nm

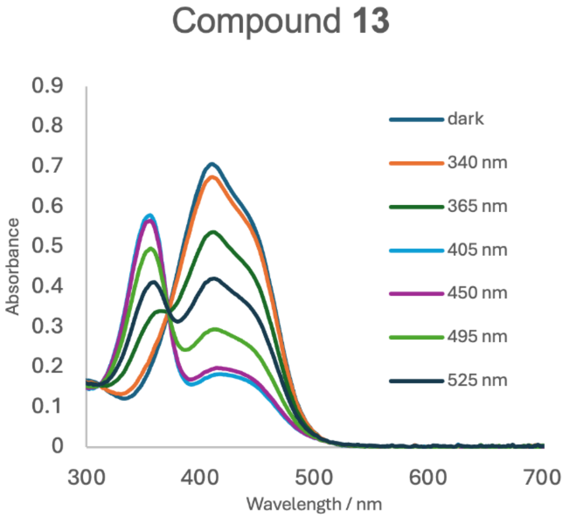
Compound 13
Absorbance
Wavelength / nm
0
0.1
0.2
0.3
0.4
0.5
0.6
0.7
0.8
0.9
300
400
500
600
700
dark
340 nm
365 nm
405 nm
450 nm
495 nm
525 nm

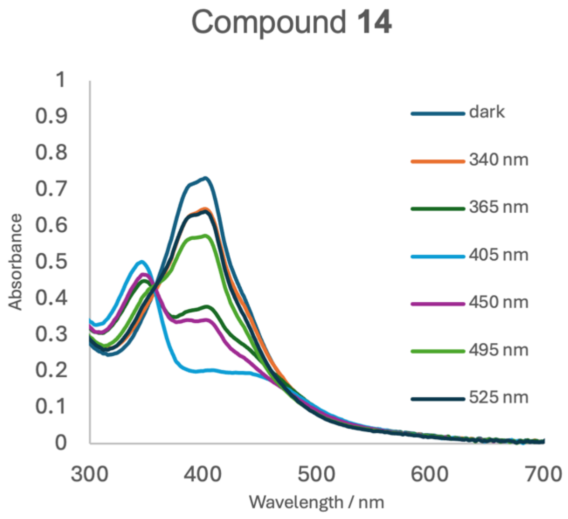
Compound 14
Absorbance
Wavelength / nm
0
0.1
0.2
0.3
0.4
0.5
0.6
0.7
0.8
0.9
1
300
400
500
600
700
dark
340 nm
365 nm
405 nm
450 nm
495 nm
525 nm

Figure S1: UV-vis spectra of the studied aryl azopyrrole photoswitches in dark states and after irradiation with LEDs of various wavelengths. Sample concentrations were 25 μM (Compound **11** and **12**) and 50 μM (**TRPswitch-C**, **10**, **13** and **14**).

Photostationary state (PSS) determination

PSS ratios were determined according to the method published by Fischer.[3] The described method allows the calculation of the photostationary states of systems that follow A ⇌ B when only A(A = *E*-isomer) is known.[4] It is assumed that the ratio of quantum yields of the forward and backward photoisomerization reaction is independent of the irradiation wavelength which allowed the calculation of the PSS for all recorded spectra/wavelengths.[4,5]

Table S1: PSS calculations according to Fischer's method.[3] Sample concentration were 25 μM (Compound **11** and **12**) and 50 μM (**TRPswitch-C**, **10**, **13** and **14**).

Compound	PSS 340 nm	PSS 365 nm	PSS 405 nm	PSS 450 nm	PSS 495 nm	PSS 525 nm
**TRPswitch C**	10% *Z*	29% *Z*	82% *Z*	81% *Z*	26% *E*	39% *E*
**10**	9% *Z*	25% *Z*	68% *Z*	69% *Z*	37% *E*	54% *E*
**11**	8% *Z*	24% *Z*	76% *Z*	80% *Z*	27% *E*	40% *E*
**12**	8% *Z*	26% *Z*	81% *Z*	81% *Z*	29% *E*	46% *E*
**13**	5% *Z*	25% *Z*	77% *Z*	75% *Z*	40% *E*	58% *E*
**14**	6% *Z*	25% *Z*	37% *Z*	73% *E*	89% *E*	94% *E*

Thermal isomerization kinetics

Thermal isomerization kinetics were directly recorded at 25 °C in an Agilent Cary 60 UV-vis spectrometer utilizing the "kinetics" function of the system. The absorption of any irradiated samples was recorded at 400 nm (410 nm for **13**) until a plateau was reached (back-isomerization achieved). The obtained absorption data was plotted against time and fitted with an exponential model (Figure S2; using Origin Pro) which allowed the calculation of the rate constant from the obtained exponential function. The half-life was then calculated using equation 1 below (Table S2).

$$t_{\frac{1}{2}} = \frac{\ln(2)}{k} \qquad (1)$$

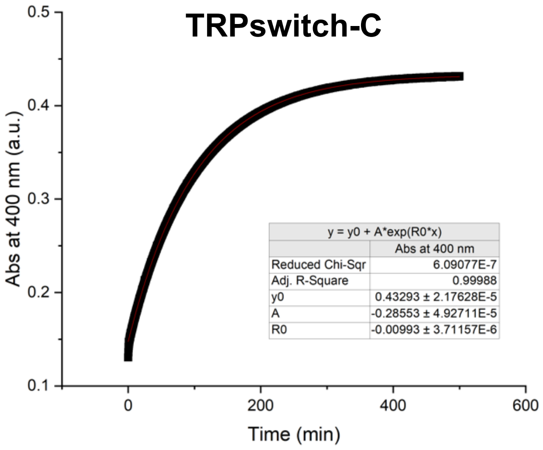

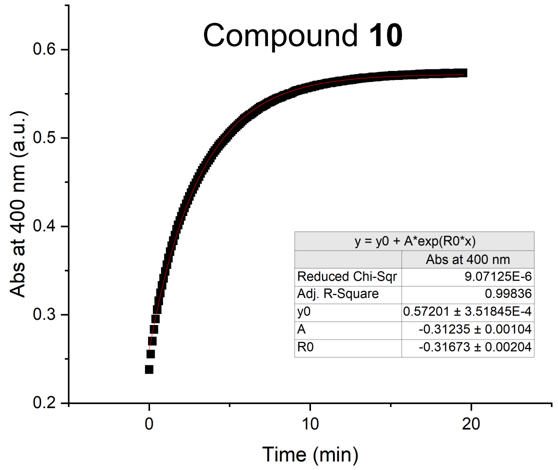

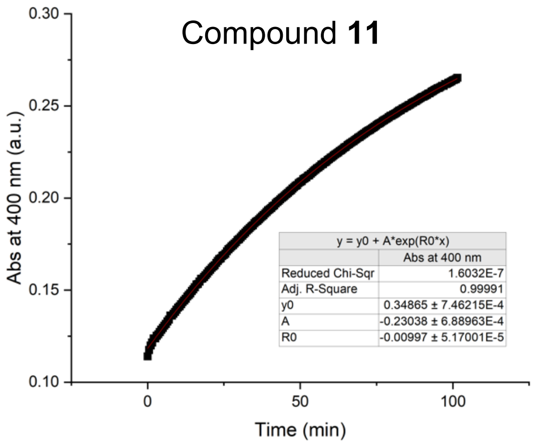

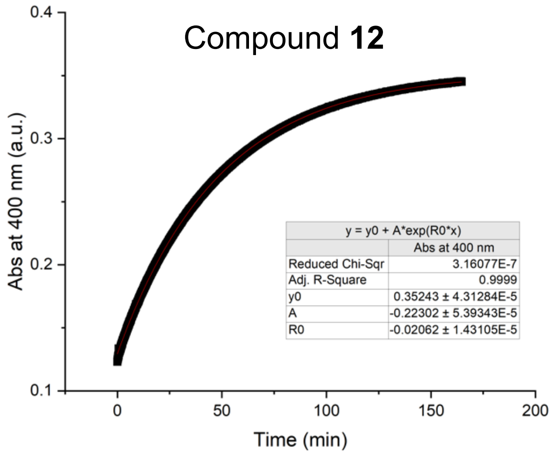

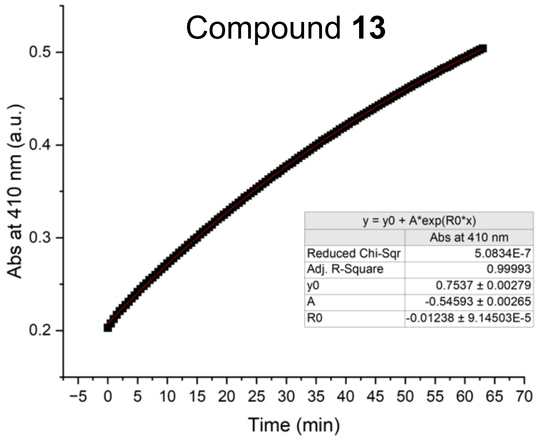

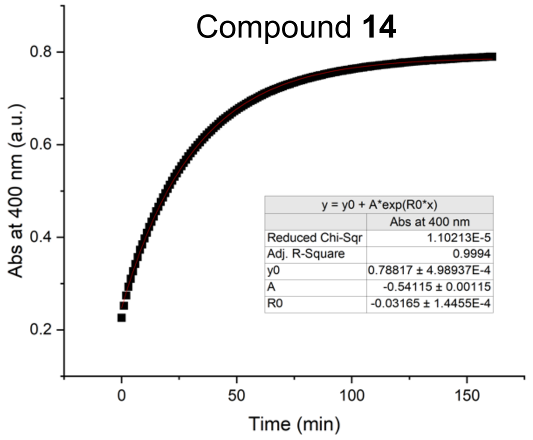

Figure S2: The recorded absorption was plotted against time and fitted to an exponential fit to obtain the rate constants of the back-isomerisation reactions.

Table S2: Calculated half-lives in minutes, according to equation 1.

Compound	Half-life (DMSO, 25 °C)
**TRPswitch-C**	69.80 mins
**10**	2.19 mins
**11**	69.52 mins
**12**	33.62 mins
**13**	55.99 mins
**14**	21.90 mins

Animal Husbandry

Wild-type TuAB zebrafish (*Danio rerio*), *trpa1b* mutants[6] or *trpv1* mutants (ZIRC ZL8929.19) were maintained and bred under normal conditions (28°C and 14/10h light/dark cycle). Larvae were collected from group matings and used for experiments. Larvae were kept in 10 cm petri dishes with 10 mM HEPES (Sigma-Aldrich, cat. no. H3375-100G) buffered E3 at 28°C with a 14/10 light/dark cycle. The maintenance of adults, collection of larvae and all experimental procedures were performed in accordance with protocols approved by the Medical College of Wisconsin's Institutional Animal Care and Use Committee (IACUC).

Light Induced Motion Response Assay

The light induced motion response assay was performed as previously described[7] with some modifications. Briefly, groups of three 3 days post fertilization (dpf) ZF larvae were placed in wells of a clear bottom black 96 well plate (Corning 3631) with 150 µl of E3. All compounds were dissolved in DMSO

(Thermo Fisher Scientific cat. no. D12345) at a working stock concentration of 2 mM. DMSO or compounds were added into wells in the dark under red light at 1% or 20 µM respectively (1:100 dilution). In all experiments, fish treated with TRPswitch-C or any of its derivatives were exposed to 1% DMSO, matching the final concentration used for the DMSO control fish. Larvae were incubated in the dark for 1 hr at 28.5°C prior to assay. Using an inverted compound Ti2 Nikon microscope with a 2x/0.10 NA air objective (Nikon) and NIS Elements acquisition software (Nikon), the assay was run via the JOBS feature. An illumination sequence was created with the NIS Elements software to control when wells were stimulated with light as described below. A spectra III light engine (Lumencor) and digital micromirror device (DMD) pattern illuminator (Mightex polygon 1000) were used for precise stimulation of one well at a time with 390nm light at 14.08 µW/mm^2, 440nm light at 5.89 µW/mm^2 or 475 nm light at 14.46 µW/mm^2. Wells were repeatedly stimulated three times for 1s with 390 nm, 440 nm or 475 nm light. Brightfield imaging was performed before and after each stimulation. A red filter was placed in the light path for bright field to avoid converting compounds when the stimulation light is not on. A Texas Red/mCherry longpass filter (cat. no. 49017; Chroma) along with a neutral density filter (UVND 1.0; Chroma) were used during light stimulation to decrease light intensity and reflect light to the sample for imaging. Frames were acquired using a Prime 95B sCMOS camera (Teledyne Photometrics) every 50ms with 2x2 binning and 16-bit depth. Motion from the resulting video was analyzed using NIS-Elements GA3 software as described below. For testing the cross activity of the compound on Trpa1b ohnolog, DNA plasmid *ngn1:zTrpa1b-2A-mCherry* (Addgene Plasmid #106425) or *ngn1:hTRPA1-2A-EGFP* construct[8] were used. Transient mosaic expression of the constructs in Rohon-Beard neurons was achieved by injecting 3 nL of a solution containing 6 ng/µL of DNA plasmid and 6.5 ng/µL *in vitro* transcribed (Ambion) Tol2 transposase mRNA into the cytoplasm of one-cell stage *trpa1b* mutant embryos. Larvae were screened at 2 dpf for fluorescent expression. The light induced motion

response assay was performed as described above, except that fish were tested individually in wells containing a final volume of 60 μl.

Analysis

Results from the light induced motion response assay is expressed in the form of a motion index. NIS Elements GA3 software was used to create a unique script that calculated the motion index of each well tested. The pixel intensity of consecutive frames was subtracted from each other (i.e. frame 1 minus frame 2, frame 2 minus frame 3, etc.) and the results were converted into an absolute value for ease of data interpretation. Thresholding based on differences in pixel intensity was performed to identify regions of the image where there was fish movement. The pixel intensities within the threshold area were added together to generate the motion index for a single frame. All the frames within the first 5s before or after light illumination were then averaged together to generate the average motion index value which is used to represent the motion of each well.

Heart experiments

Heartbeat interruption experiments were performed *in vivo* on *Tg(cmlc2:Trpa1b-2A-EGFP)*[8] ZF. At 3 dpf, larvae were incubated in either 1% DMSO or 20 μM TRPswitch-C (1:100 dilution) in the dark for 1 hr at 28.5°C prior to the assay. Immediately before the assay, larvae were anesthetized with 0.2 mg/mL tricaine (Sigma, A5040) and mounted laterally in 1% low melt agarose (LMA; Genesee Scientific, cat. no. 20-104) with the right side of the larvae facing the glass-bottom plate (Cellvis, cat. no. D35-20-0-N). Mounting was performed under room light only to prevent photoconversion of the compound. Larvae were then incubated in either 1% DMSO or 20 μM TRPswitch-C in E3 without tricaine for 10 mins before imaging. The assay was conducted using an inverted Nikon Ti2 compound microscope equipped with a 20x/0.75 NA air objective and NIS Elements acquisition software (Nikon). Illumination was

controlled via the Illumination sequence feature. A spectra III light engine (Lumencor) and a digital micromirror device (DMD) pattern illuminator (Mightex polygon 1000) were used to stimulate only the heart with 390 nm or 555 nm light in a circular region with a radius of 88 μm. The illumination sequence consisted of repeated cycles as follows: 10s of brightfield (BF) imaging, 10s of 390 nm stimulation, 5s of BF imaging, 10s of 555 nm stimulation, and 10s of BF imaging. The sequence was looped 3 times. A red filter was placed in the light path during BF imaging to avoid converting TRPswitch-C when the stimulation light was off. A Texas Red/mCherry longpass filter (cat. no. 49017; Chroma) was used during light stimulation to reflect light onto the sample for imaging. Frames were acquired every 50 ms using a Prime 95B sCMOS camera (Teledyne Photometrics) with 2x2 binning and 16-bit depth. Ventricle width measurements were performed using the ImageJ "measure" function, selecting the widest point of the ventricle every 100 ms throughout the video. Heartbeat frequency was manually counted for all videos.

Statistical analyses

All results are expressed as mean ± SEM. Statistics analysis for Figure 2D, Figure 5A, and Figure 6D was performed using a two-way ANOVA; for Figure 3, Figure 4A and B, and Figure 5B an ordinary one-way ANOVA was used. The criterion for statistical significance was $p < 0.1234$. All statistical analyses were performed using Prism (GraphPad Software).

References

1 K. W. Shimkin, P. G. Gildner and D. A. Watson, Copper-Catalyzed Alkylation of Nitroalkanes with alpha-Bromonitriles: Synthesis of beta-Cyanonitroalkanes, *Org. Lett.,* 2016, **18**, 988–991.

2 S. K. Pagire, A. Hossain and O. Reiser, Temperature Controlled Selective C-S or C-C Bond Formation: Photocatalytic Sulfonylation versus Arylation of Unactivated Heterocycles Utilizing Aryl Sulfonyl Chlorides, *Org. Lett.,* 2018, **20**, 648–651.

3 E. Fischer, Calculation of photostationary states in systems A ⇌ B when only A is known, *J. Phys. Chem.,* 1967, **71**, 3704–3706.

4 N. K. Singer, K. Schlogl, J. P. Zobel, M. D. Mihovilovic and L. Gonzalez, Singlet and Triplet Pathways Determine the Thermal Z/E Isomerization of an Arylazopyrazole-Based Photoswitch, *J. Phys. Chem. Lett.,* 2023, **14**, 8956–8961.

5 D. V. Berdnikova, Design, synthesis and investigation of water-soluble hemi-indigo photoswitches for bioapplications, *Beilstein J. Org. Chem.,* 2019, **15**, 2822–2829.

6 D. A. Prober, S. Zimmerman, B. R. Myers, B. M. J. McDermott, S. Kim, S. Caron, J. Rihel, L. Solnica-Krezel, D. Julius, A. J. Hudspeth and A. F. Schier, Zebrafish TRPA1 channels are required for chemosensation but not for thermosensation or mechanosensory hair cell function, *J. Neurosci.,* 2008, **28**, 10102–10110.

7 P. Lam, A. R. Thawani, E. Balderas, A. J. P. White, D. Chaudhuri, M. J. Fuchter and R. T. Peterson, TRPswitch-A Step-Function Chemo-optogenetic Ligand for the Vertebrate TRPA1 Channel, *J. Am. Chem. Soc.,* 2020, **142**, 17457–17468.

8 P. Lam, S. K. Mendu, R. W. Mills, B. Zheng, H. Padilla, D. J. Milan, B. N. Desai and R. T. Peterson, A high-conductance chemo-optogenetic system based on the vertebrate channel Trpa1b, *Sci. Rep.,* 2017, **7**, 11839.
